# The epigenomic landscape of African rainforest hunter-gatherers and farmers

**DOI:** 10.1038/ncomms10047

**Published:** 2015-11-30

**Authors:** Maud Fagny, Etienne Patin, Julia L. MacIsaac, Maxime Rotival, Timothée Flutre, Meaghan J. Jones, Katherine J. Siddle, Hélène Quach, Christine Harmant, Lisa M. McEwen, Alain Froment, Evelyne Heyer, Antoine Gessain, Edouard Betsem, Patrick Mouguiama-Daouda, Jean-Marie Hombert, George H. Perry, Luis B. Barreiro, Michael S. Kobor, Lluis Quintana-Murci

**Affiliations:** 1 Institut Pasteur, Unit of Human Evolutionary Genetics, Paris 75015, France; 2 Centre National de la Recherche Scientifique, URA3012, Paris 75015, France; 3 Université Pierre et Marie Curie, Cellule Pasteur UPMC, Paris 75015, France; 4 Centre for Molecular Medicine and Therapeutics, Child and Family Research Institute and Department of Medical Genetics, University of British Columbia, Vancouver, Canada BC V5Z 4H4; 5 INRA, UMR AGAP, Montpellier 34060, France; 6 IRD-MNHN, Sorbonne Universités, UMR208, Paris 75005, France; 7 CNRS, MNHN, Université Paris Diderot, Sorbonne Paris Cité, Sorbonne Université, UMR7206, Paris 75005, France; 8 Institut Pasteur, Unité d'Epidémiologie et Physiopathologie des Virus Oncogènes, Paris 75015, France; 9 Faculty of Medicine and Biomedical Sciences, University of Yaoundé I, BP1364 Yaoundé, Cameroon; 10 Laboratoire Langue, Culture et Cognition (LCC), Université Omar Bongo, BP 13131 Libreville, Gabon; 11 CNRS UMR 5596, Université Lumière-Lyon 2, Lyon 69007, France; 12 Departments of Anthropology and Biology, Pennsylvania State University, University Park, Pennsylvania 16802, USA; 13 Université de Montréal, Centre de Recherche CHU Sainte-Justine, Montréal, Canada H3T 1C5

## Abstract

The genetic history of African populations is increasingly well documented, yet their patterns of epigenomic variation remain uncharacterized. Moreover, the relative impacts of DNA sequence variation and temporal changes in lifestyle and habitat on the human epigenome remain unknown. Here we generate genome-wide genotype and DNA methylation profiles for 362 rainforest hunter-gatherers and sedentary farmers. We find that the current habitat and historical lifestyle of a population have similarly critical impacts on the methylome, but the biological functions affected strongly differ. Specifically, methylation variation associated with recent changes in habitat mostly concerns immune and cellular functions, whereas that associated with historical lifestyle affects developmental processes. Furthermore, methylation variation—particularly that correlated with historical lifestyle—shows strong associations with nearby genetic variants that, moreover, are enriched in signals of natural selection. Our work provides new insight into the genetic and environmental factors affecting the epigenomic landscape of human populations over time.

## Abstract (web summary)

Genetic and environmental factors affect genome-wide patterns of epigenetic variation. Here, the authors show that while current habitat and historical lifestyle impact the methylome of rainforest hunter-gatherers and sedentary farmers, the biological functions affected and the degree of genetic control differ.

## Graphical abstract

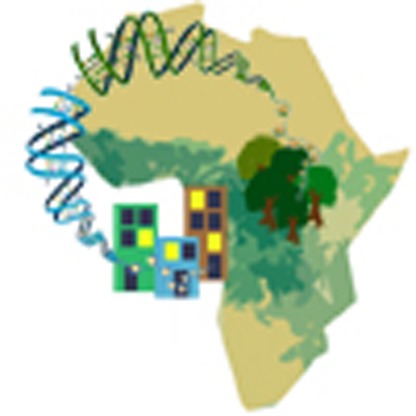

Africa is the birthplace of modern humans and a region of extensive genetic, cultural, environmental and phenotypic diversity^1^. Over the past years, the increasing amounts of genomic data available have provided significant insight into African evolutionary history, including the origins of hunter-gatherers, population structure, and patterns of migration and admixture^2^,^3^,^4^,^5^,^6^,^7^,^8^,^9^,^10^. Moreover, these studies have reported evidence of selection targeting gene functions related to the changes in environment, diet and exposure to infectious disease^11^. Adding an additional layer of complexity, the study of epigenetic variation can inform the interplay between the environment and the genome, yet the epigenomic landscape of African populations remains unexplored.

DNA methylation—an important epigenetic mark that serves as biomarker for variation in gene regulation^12^,^13^—can be affected by both inherited DNA sequence variation and a broad range of environmental factors, such as nutrition, exposure to toxic pollutants and social environment^14^,^15^,^16^,^17^. Accumulating evidence indicates that a substantial portion of DNA methylation variation is accounted for by genetic variation (methylation quantitative trait loci, meQTLs)^16^,^18^,^19^,^20^,^21^,^22^, which could affect methylation levels through impaired transcription factor (TF) binding^12^,^13^. Although the role of DNA methylation in gene regulation (active or passive) and the mechanisms involved remain controversial, DNA methylation data offer a rich source of information about ongoing gene activity, and thus it can provide insight into gene functions that contribute to phenotypic variation^12^,^13^. Recent studies have shown that DNA methylation differences exist between major ethnic groups^20^,^23^,^24^,^25^, highlighting the potential contribution of epigenetic modifications to human phenotypic variation. However, these studies have mostly compared urban populations of different continental ancestries, so the relative impacts of DNA sequence variation and temporal changes in lifestyle and habitat on the human DNA methylome remain unknown.

The Central African belt provides an ideal setting in which to address this issue, as it hosts the world's largest group of active hunter-gatherers—the rainforest hunter-gatherers (RHGs, traditionally known as ‘pygmies')—as well as populations that have adopted an agrarian lifestyle (AGRs) over the last 5,000 years^26^,^27^. In addition to differing in their subsistence strategies, these two groups differ in other historical and recent aspects of their evolutionary history. The historical factors relate to the differences in demography and habitat. The ancestors of the RHGs and AGRs diverged ∼60,000 years ago^7^,^8^,^28^,^29^,^30^ and subsequently experienced population contractions and expansions, respectively^10^. These groups have also historically occupied separate ecological habitats—the ancestors of RHGs the equatorial rainforest while those of AGRs open spaces, such as savannah and grasslands^27^,^31^. More recent changes in the lifestyles and habitats of these groups are also apparent. Many RHG groups still live in the rainforest as mobile bands, whereas AGR populations now occupy primarily rural or urban deforested areas, though some AGR groups have settled in the rainforest over the last millennia^27^,^31^.

In this study, we define the genome-wide DNA methylation profiles in blood of various populations of RHG and AGR inhabiting the Central African belt to first assess the degree of inter-population variation in DNA methylation. We then explore the genomic and functional features of differentially methylated genes to obtain insight into the putative phenotypes involved. Finally, we assess the contribution of genetic variation to the DNA methylation levels observed, and search for signals of positive selection targeting genetic variants associated with methylation variation.

Here, we show that while both recent changes and historical differences in the habitat and lifestyle of RHG and AGR have had a critical impact on their patterns of DNA methylation variation, the biological functions affected strongly differ. We also show that DNA methylation variation that correlates with historical lifestyle shows strong associations with nearby genetic variants that, moreover, are enriched in signals of natural selection. The integration of these results allow us to propose a comprehensive framework of how temporal differences in lifestyle and habitat, together with the genetic variation, have impacted the epigenomic landscape of human populations.

## Results

### Population samples and genetic structure

**Figure 1:**
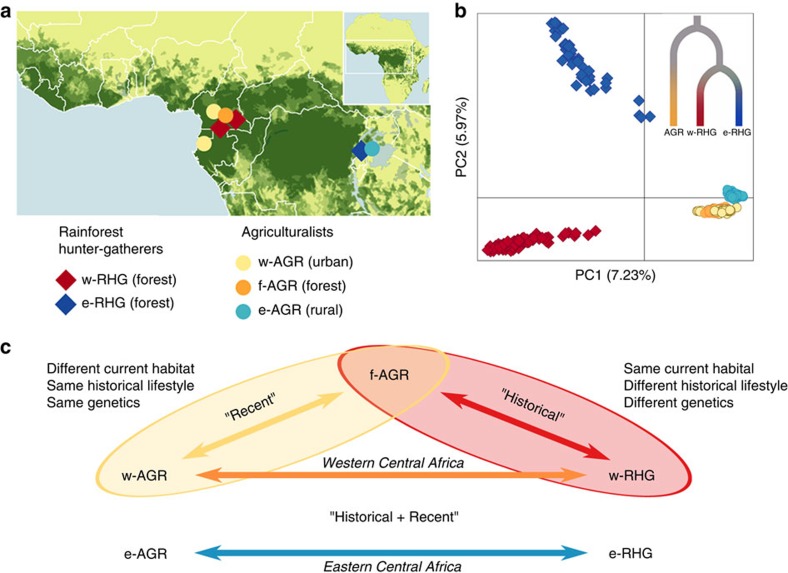
Study design and genetic structure of rainforest hunter-gatherers and farmers. (**a**) Geographic location of the sampled rainforest hunter-gatherer (RHG) and farmer (AGR) populations. (**b**) Principal component analysis (PCA) of the genotype data for the study populations, based on 456,507 independent genome-wide SNPs. The tree presented at the top right of the panel represents the branching model for these populations^7^,^8^,^28^,^29^,^30^. (**c**) Schematic representation of the different population comparisons, indicated by arrows, used for the detection of differentially methylated sites (DMS) between groups.

We investigated genome-wide genotype and DNA methylation data from a total of 362 individuals, including a group of RHGs (w-RHG, *n*=112), AGR groups occupying nearby urban deforested habitats (w-AGR, *n*=94), and an AGR group that lives and regularly practices hunting in a forested region (f-AGR, *n*=61) of the Gabon/Cameroon area (Fig. 1a; Table 1). To compare our results with an independent set of samples, we also studied RHGs and AGRs living in the eastern part of the Central African belt (e-RHG, *n*=47 and e-AGR, *n*=48, from Uganda). We first investigated the global genetic structure of the studied populations using genome-wide SNP (single nucleotide polymorphism) data. Principal component analysis (PCA) clearly reflected their history of population divergence^7^,^8^,^28^,^29^,^30^. The largest differences were observed between RHG and AGR populations, regardless of their geographic location, followed by the more recent split between the western and eastern Central African RHG groups (Fig. 1b).

### Processing genome-wide DNA methylation data

We characterized DNA methylation variation in whole blood-derived samples using the Illumina 450 K array, which interrogates more than 485,000 sites across the genome. After normalization and filtering, including the removal of probes containing genetic variants at a frequency higher than 1% in the populations studied, we retained 365,886 probes in 352 individuals (Methods). Samples showed both high reproducibility and expected DNA methylation profiles across genomic regions, with sites near gene promoters being less methylated than those located in gene bodies and intergenic regions (Supplementary Note 1; Supplementary Fig. 1).

We next sought to correct methylation values (*M*-values) for known biological and technical potential confounders, including gender, age and heterogeneity in blood cell composition. We thus estimated ages for all samples, and compared predicted and declared ages for individuals in which chronological age was reliably ascertained (*N*=256, Pearson's *R*=0.84; Supplementary Fig. 2; Supplementary Note 2), confirming the accuracy of the epigenetic clock model^32^. Similarly, we estimated the proportions of different blood cell types in all samples, using a predictive model based on a subset of DNA methylation probes^33^, which were removed from all subsequent analyses, yielding a final set of 365,401 probes. These predicted values showed strong correlations with observed proportions of blood cell subtypes, which were determined in a subset of samples (*N*=66) by fluorescence-activated cell sorting (Pearson's *R*: 0.48–0.57; Supplementary Fig. 3; Supplementary Note 3). Thus, gender, estimated ages and cell subtype heterogeneity across populations were used to adjust *M*-values for all subsequent analyses, including PCA, the estimation of differentially methylated sites and the mapping of methylation quantitative trait loci.

### Population differences in DNA methylation profiles

When performing PCA using all samples, while age and cell counts strongly correlated with the first 10 PCs using unadjusted *M*-values, the subsistence strategy (RHG versus AGR) and geographic location (western versus eastern Central Africa) of the populations were the only factors associated with the first 10 PCs using adjusted *M*-values (Supplementary Fig. 4; Supplementary Table 1). Because of technical variables associated with differences in sample collection and DNA processing between the western and eastern African samples, one cannot entirely rule out that the observed geographic differences are due to technical factors. To understand the relationship between DNA methylation variation and differences in subsistence strategies and habitat, we thus performed all subsequent population comparisons within each geographic region separately.

**Figure 2:**
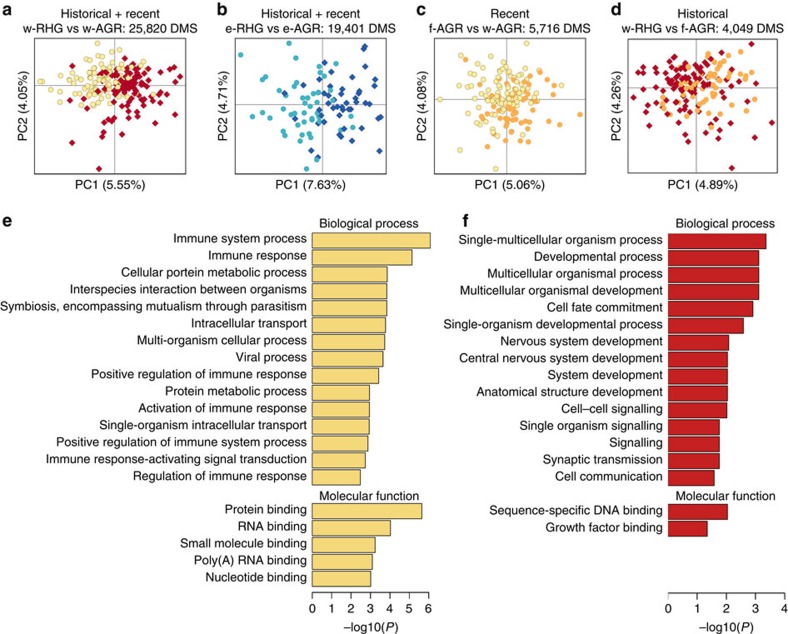
DNA methylation profiles and functional differentially methylated regions. (**a**–**d**) PCA of genome-wide DNA methylation profiles for the different population comparisons. (**e**,**f**) Gene ontology (GO) enrichment analysis for (**e**) recent DMS and (**f**) historical DMS. The top GO categories for biological processes and molecular functions are shown, together with the log-transformed FDR-adjusted enrichment *P* values.

We compared DNA methylation variation between populations differing in genetic background, historical lifestyle and current habitat—the RHG and AGR groups living in the rainforest and rural/urban areas, respectively (Fig. 1c). PCA clearly separates the RHG and AGR groups on PC1, in both western (*P*=9.9 × 10^−15^) and eastern (*P*=5.7 × 10^−11^) Central Africa (Fig. 2a,b; Supplementary Fig. 5). We identified 25,820 differentially methylated sites (DMS; located across 8,803 genes) between w-RHG and w-AGR, and 19,401 DMS (located across 6,288 genes) between e-RHG and e-AGR (false discovery rate (FDR)<0.01). Interestingly, when comparing the western and eastern settings, we detected an overlap of 6,844 sites (located across 2,528 genes) differentially methylated in the same direction—corresponding to 96% of the overlapping DMS (resampling *P*<10^−7^). Collectively, these findings attest to strong, shared differences in DNA methylation between RHG and AGR groups, regardless of their geographic location.

### Impact of habitat and lifestyle changes on DNA methylation

To distinguish the respective effects on DNA methylation of recent changes in habitat from historical differences in lifestyle and genetics of these groups, we next compared populations with a common historical lifestyle and genetic background but different recent habitats, specifically the forest f-AGR and the urban w-AGR (Fig. 1c). The observed patterns of DNA methylation variation were accounted for primarily by the habitat in which the populations live (PC1 *P*=3.5 × 10^−4^; Fig. 2c), highlighting the important role of current habitat in determining global DNA methylation profiles. We found 5,716 DMS (located across 3,550 genes) between the two groups, which we termed ‘recent DMS'. The differential methylation in the same direction of 3,304 of these recent DMS (corresponding to 99% of the overlapping DMS, resampling *P*<10^−7^; 2,146 genes) between the more distantly related w-RHG and w-AGR provided strong evidence in favour of the methylation status at these shared DMS being determined by recent changes in habitat independently of genotypic differences.

Focusing on populations with different historical lifestyles and genetic backgrounds but with the same current habitat (f-AGR and w-RHG in the Central African rainforest, Fig. 1c), PCA also tended to separate the samples with respect to their population identity (PC1 *P*=2.4 × 10^−5^; Fig. 2d). We found 4,049 DMS (located across 2,128 genes) between these groups, which we termed ‘historical DMS'. Notably, historical DMS presented larger absolute differences in mean DNA methylation levels between populations (|Δβ|, using here β-values instead of *M*-values, see ref. 34) than recent DMS. In particular, the proportion of DMS for which |Δβ| values are >5% was higher for historical than for recent DMS (*P*<10^−16^; Supplementary Fig. 6a,b). These historical DMS showed no significant overlap with the recent DMS described above (only 52 DMS were shared). The set of historical DMS identified thus reflects DNA methylation variation related to the historical differences in lifestyle and habitat characterizing the RHG and AGR groups.

### Genomic features of differentially methylated regions

To understand the putative functional implications of DMS, we first localized them across distinct genomic regions. We found that recent DMS were enriched in sites located in gene bodies and distal promoters, while historical DMS were preferentially located around the transcription start sites (TSS), 5′-UTR (untranslated region) and first exon regions (Supplementary Fig. 7a,c). We next mapped DMS to histone modification peaks from peripheral blood mononuclear cells (PBMCs) as mapped by the ENCODE project^35^. We found that both recent and historical DMS mapped in excess to H3K4me1 modification peaks (32% for both DMS sets versus 20% expected) (Supplementary Fig. 7b,d). Notably, the recent DMS that were hypermethylated in f-AGR were further enriched in H3K4me3 peaks (57% versus 27%), while the historical DMS that were hypermethylated in w-RHG were enriched in H3K27me3 (32% versus 12%).

Finally, we explored the colocalization of DMS with TF-binding sites (Methods). We found that recent DMS were significantly enriched in binding sites of TFs related to cell differentiation, proliferation and development, but also to immune regulation (NFIL3, IRF1 and GATA3), and fatty acid storage and glucose metabolism (HNF1A, RORA and NR1H2::RXRA) (Supplementary Table 2). Conversely, historical DMS, particularly those that were hypermethylated in RHG, were preferentially overlapping binding sites of TF involved in developmental processes (TFAP2A and NHLH1). Collectively, these findings indicate that recent and historical DMS not only represent independent sets, but also are located in distinct genomic regions that contain different TF-binding sites, suggesting that they are associated to regulatory features related to different biological functions.

### Biological functions of recent and historical DMS differ

We investigated the relevance of recent and historical DMS for explaining phenotypic diversity by exploring whether differentially methylated genes in each set were enriched in gene ontology categories or in genes reported to be associated with traits or diseases by genome-wide association studies (GWAS). We found that genes containing recent DMS were enriched in categories largely associated with immune response, host–pathogen interactions and various cellular processes (Fig. 2e; Supplementary Table 3). Consistently, recent DMS genes were enriched in genes reported by GWAS (FDR-corrected resampling *P*<8.1 × 10^−3^), including autoimmune disorders, such as vitiligo (20 genes associated versus 10.1 expected, *P*=0.045) and systemic lupus erythematosus (19 genes associated with versus 9.2 expected, *P*=0.028).

Conversely, genes overlapping historical DMS were enriched in functions almost exclusively related to developmental processes, including multicellular organismal development, anatomical structure development, or growth factor binding (Fig. 2f; Supplementary Table 3). In contrast to recent DMS, historical DMS genes were not enriched in genes reported by GWAS. We also found that 1,302 historical DMS (699 genes) overlapped with the DMS detected in western (w-AGR versus w-RHG) and eastern (e-RHG versus e-AGR) comparisons, in the same direction (corresponding to 99% of the overlapping DMS, resampling *P*<10^−7^), despite the splitting of the RHG groups ∼20,000 years ago^8^,^28^,^30^. This common set of historical DMS was again enriched in functions primarily related to development (Supplementary Table 4). We thus identified a gene set in which epigenomic variation reflected differences in the lifestyle and habitat, as well as in genetic background, of RHGs and AGRs, regardless of their geographic location.

Because recent DMS were found to be particularly enriched in functions related to immune processes, we next evaluated the extent to which potential variability in blood cell proportions, despite our adjustments for cell count heterogeneity (Supplementary Note 3), may still affect our findings. No major differences in immune cell counts were observed between the populations compared (Supplementary Fig. 8; Supplementary Note 4). Furthermore, when using a ‘filtered' data set, in which we removed a set of 51,386 probes that have been shown to correlate with cell counts by an independent study^36^, we found that the biological functions associated with recent and historical DMS clearly differed and were primarily associated with host–pathogen interactions/cellular processes and development, respectively (Supplementary Table 5; Supplementary Note 4), confirming the results obtained using the global data set.

### Genetic contribution to DNA methylation variation

To assess the contribution of genetic variation to the DNA methylation levels, we mapped meQTLs, focusing our analyses on SNPs located in *cis* within a 200-kb window around the target site (Methods; Supplementary Fig. 9). We identified 45,916 DNA methylation sites (∼13% of all sites) associated with a nearby meQTL, in at least one population, with a FDR set to 1%. The majority of meQTLs (∼90%) were shared across populations, with only 1,283 and 500 meQTLs detected exclusively in the RHG and AGR groups, respectively. Such extensive sharing of meQTLs reflects the closer genetic proximity of the populations studied here and the use of a different cellular model, with respect to previous studies^23^,^25^ (Supplementary Fig. 10; Supplementary Table 6; Supplementary Note 5).

**Figure 3:**
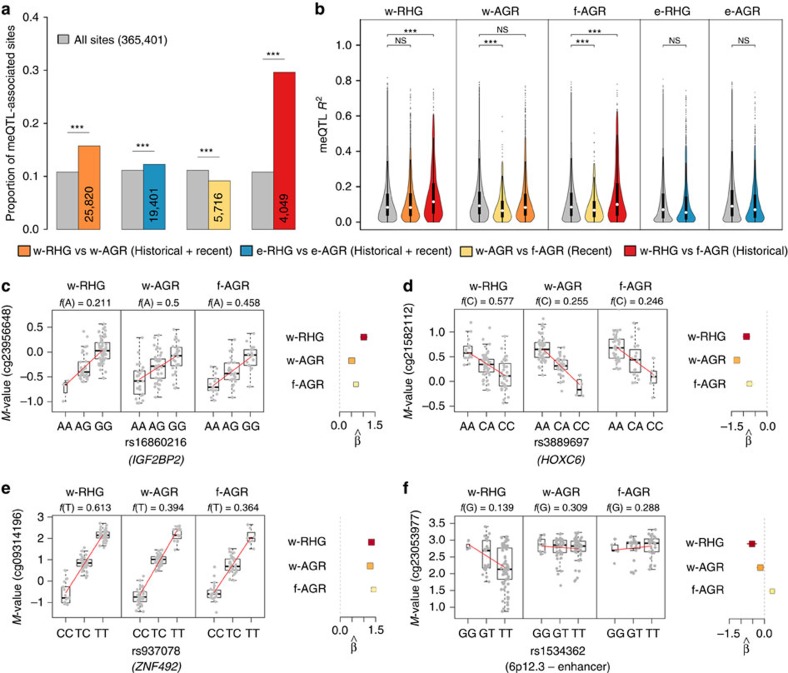
Contribution of genetic variation to the DNA methylation levels. (**a**) Proportion of methylation sites that are associated with a nearby genetic variant (in grey) and among different DMS sets (in colour). The numbers in the bars correspond to the total number of DMS per population comparison. *P* values were calculated by resampling. (**b**) Proportion of the variance of DNA methylation explained by nearby genetic variants (*R*^2^) for the various meQTL sets, in each population. The *P* values (Mann–Whitney *U*-test) obtained indicate a significant skew in the *R*^2^ distribution of the various meQTL–DMS sets (in colour) with respect to that of all meQTLs (in grey) in the corresponding population. *R*^2^ values are higher for meQTLs associated with historical DMS (11.5% (10.7–12.3%) and 10.0% (8.9–11.2%) in w-RHG and f-AGR, respectively) than for those related to recent DMS (6.5% (5.7–7.2%) and 6.8% (6.1–7.4%) in w-AGR and f-AGR, respectively). NS, not significant, *P<0.05, **P<0.01, ***P<0.001. (**c**–**f**) Examples of meQTLs detected in this study. The three boxplots on the left represent the distribution of *M*-values as a function of genotype. The minor allele frequency of each meQTL is presented for each population. Red lines indicate the fitted linear regression model for *M*-value ∼ genotype for each population. The forest plots on the right represent the estimated β, corresponding to the slope of the linear regression, for each population. (**c**–**e**) meQTLs detected in all populations but presenting different allelic frequencies between RHG and AGR groups. The mean *F*_ST_ values between w-RHG and f-AGR/w-AGR groups for the SNPs concerned were higher (0.15, 0.19 and 0.10, respectively) than that observed genome wide (*F*_ST_<0.03). (**f**) Population-specific meQTL, where the SNP rs1534362 is associated with methylation differences in the enhancer region at 6p12.3 only in RHGs.

We next tested the potential enrichment of differentially methylated regions in associations with genotype variants, with respect to all DNA methylation sites. We found a moderate enrichment in DMS characterizing the western (16%, (odds ratio) OR=1.5, s.e.=0.02; resampling *P*<10^−7^) and eastern comparisons (12%, OR=1.1, s.e.=0.02; resampling *P*=1.2 × 10^−2^), where populations differ in both historical and recent lifestyles and habitats (Fig. 3a). Furthermore, historical DMS were strongly enriched in meQTLs (30%, OR=3.5, s.e.=0.03; resampling *P*<10^−7^), whereas recent DMS were depleted in these associations (9%, OR=0.80, s.e.=0.05; resampling *P*<10^−7^). These findings were replicated using the ‘filtered' data set (Supplementary Note 4), indicating that the potential presence of blood cell heterogeneity is unlikely to account for these observations.

We also found that the proportions of DMS associated with meQTLs were systematically higher in historical than in recent DMS, irrespective of the mean differences in DNA methylation levels between populations (Supplementary Fig. 6c). In addition, the proportion of the variance of DNA methylation accounted for by meQTLs (*R*^2^) was higher for meQTLs associated with historical DMS (∼11%) than for meQTLs related to recent DMS (6.6%), the *R*^2^ values obtained being significantly higher and lower, respectively, than for all meQTLs (Fig. 3b). Consistent with all our previous observations, historical DMS were more strongly associated with genotypic differences, which had also a larger effect, than the remaining sets of DMS.

Two scenarios can explain the observed associations between historical DMS and DNA sequence variants. In the large majority of cases (∼96%), DNA methylation differences were accounted for by meQTLs detected in all populations but with differences in allelic frequency between the RHG and AGR groups (Fig. 3c–e; see Supplementary Fig. 11 for more examples). More rarely (∼4%), genetic variants appeared to correlate with DNA methylation differences only in some populations, indicating interactions with other genetic variants and/or the environment (G × G or G × E interactions; Fig. 3f).

To validate our findings and evaluate the possibility that despite our stringent filtering criteria (Methods), unknown genetic variants in the methylation probe sequence may still drive some of these associations, we compared our array findings to bisulfite pyrosequencing of a selected group of DMS associated with a meQTL (that is, *IGF2BP2*, *HOXC6*, *ZNF492*, 6p12.3, *DOCK1*, *COL23A1*, *RORA* and *ADAM28*). We observed, in all cases, a very good correlation between the DNA methylation levels measured by pyrosequencing and the array (Pearson's *R*=0.74–0.94) as well as a good agreement between the two methods (Supplementary Figs 12 and 13). Our results were verified by an independent method, where we confirmed both the differences in methylation levels and the association with meQTLs for these probes, thus suggesting that unfiltered genetic variation on the 450 K array is unlikely to have contributed to the global patterns of DNA methylation observed.

### Signals of positive selection targeting meQTLs

**Figure 4:**
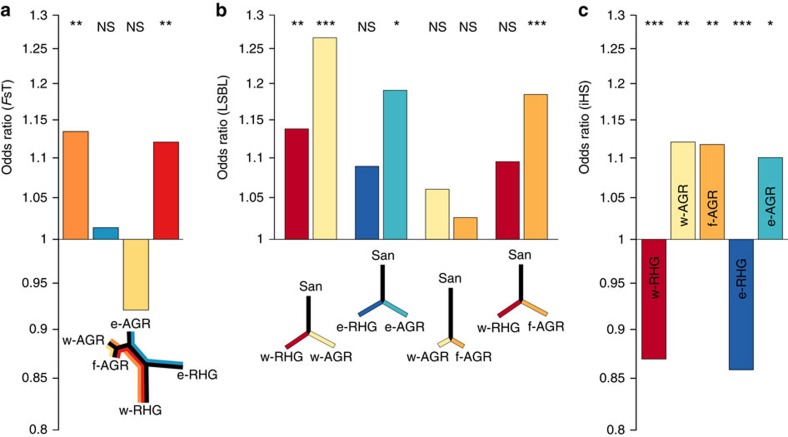
Selection signals at genetic variants associated to DNA methylation levels. (**a**,**b**) Odds ratios measuring the enrichment in high (**a**) *F*_ST_ and (**b**) LSBL values among meQTLs, with respect to the remainder of genome-wide SNPs located in a 20-kb window surrounding each methylation probe, in the different population comparisons. *P* values were calculated using a Cochran–Mantel–Haenszel test, stratified by derived allele frequencies. The colours in the plots correspond to the (**a**) population comparisons and (**b**) genetic distances shown in the schematic trees below each plot. (**c**) Odds ratios measuring the enrichment in high |iHS| values for the different meQTL data sets (in colour). *P* values were estimated using a *χ*^2^-test. For *F*_ST_, LSBL and |iHS|, we considered only SNPs with an LD *r*^2^<0.8. NS, not significant, *P<0.05, **P<0.01, ***P<0.001.

Finally, we explored the adaptive significance of meQTLs using three metrics that detect positive selection signals: *F*_ST_, which compares the variance of allele frequencies within and between populations^37^; the locus-specific branch length (LSBL), which uses pairwise calculations of *F*_ST_ from three or more populations to detect population-specific changes in allele frequency^38^; and the integrated haplotype score (iHS), which is based on the degree of extended haplotype homozygosity^39^. We found that meQTLs were significantly enriched in high *F*_ST_ and LSBL values with respect to the remainder of genome-wide SNPs located in the vicinity of a methylation probe, in nearly all population comparisons involving the RHG and AGR groups (Fig. 4a,b). In addition, LSBL analysis revealed that the enrichment in signals of RHG–AGR population differentiation detected at meQTLs is particularly observed in AGR populations. Likewise, meQTLs were significantly enriched in high |iHS| among AGR groups, suggesting more recent events of positive selection targeting regulatory variation in these groups (Fig. 4c). Collectively, these findings suggest that positive selection has targeted DNA sequence variants that influence—directly or indirectly—variation in DNA methylation.

## Discussion

Dissecting the means by which populations have responded, and conceivably adapted, to environmental cues associated with changes in subsistence strategies and ecological habitats is key to understand the mechanisms underlying natural phenotypic variation. Studies of genetic adaptation of African populations, including hunter-gatherers such as ‘pygmies', Hadza, Sandawe and San, have detected selection signals in genes related to morphology, diet and immune response, and shown that most of these signals are unique to each population group^1^,^5^,^7^,^40^,^41^,^42^. These studies have increased our knowledge of how populations might have genetically adapted to their respective environments. However, the impact that temporal changes in subsistence strategies and habitat, together with genetic diversity, have on epigenetic variation remains unexplored, despite it can inform about additional mechanisms of human responses to environmental challenges. Our findings show that recent and historical changes in habitat and lifestyle have both critical impacts on DNA methylation variation, with differences in the functions affected and the degree of genetic control.

One possible limitation of our study is the measurement of DNA methylation from whole blood^36^, which could reflect population differences in the abundance of cell types, particularly when it comes to compare populations being exposed to different environmental challenges (that is, those used to detect recent DMS). Indeed, a diverse set of environmental factors, including air pollution, exposure to carcinogens and socioeconomic status, have been shown to affect DNA methylation in blood cells^16^,^43^,^44^. Environmental variables can also alter blood cell proportions, but it remains unclear whether changes in DNA methylation are the cause or the consequence of such cellular patterns^15^. Although we cannot completely rule out a partial effect of cell composition, we adopted stringent measures to control for it (Supplementary Notes 3 and 4). These analyses support the conclusion that variability in blood cell subtypes should not have a major effect on our findings (for example, replication of both the differences in biological functions between recent and historical DMS and enrichment in genetic control of historical DMS), and suggest a series of important biological implications.

First, we show that recent changes in habitat, such as those experienced by agriculturalist populations living in urban/rural areas or in the rainforest, can substantially alter the methylome of genetically homogeneous populations, indicating that most of their divergence in DNA methylation is unlikely to be explained by underlying genetic differences. Such epigenetic alterations affect principally immune functions and processes involved in host–pathogen interactions and cellular metabolism. This is consistent with previous findings based on gene expression variation in Moroccan populations, where immunity is the most altered function in urban populations, as compared with rural and nomadic groups^45^. We also find that differentially methylated regions between urban and forest-based farmers are particularly enriched in genes associated with autoimmune disorders, suggesting that urbanization likely has an influence on susceptibility to immunity-related disorders, as previously hypothesized for allergies and inflammatory bowel disease^46^,^47^. Although the underlying mechanisms remain unknown, highlighting the need of additional studies of DNA methylation variation using purified cell types and tissues, our results suggest functional links between DNA methylation variation and environmentally triggered phenotypes, owing to a combination of biotic, abiotic and cultural factors associated with increasing urbanization and modern lifestyles.

Second, when comparing rainforest hunter-gatherers and farmers who share the same forest environment—a setting that minimizes the effects that recent environmental changes have had on methylation—we find that DNA methylation differences related to historical factors mostly reside in genes with functions in developmental processes. Furthermore, such differences in DNA methylation profiles are strongly associated with nearby genetic variants, the frequency of which differs between hunter-gatherer and farmer groups. This is the case, for example, for meQTLs in genes such as *IGF2BP2*, *HOXC6* and *ZNF492* (Fig. 3c–e), which have been associated with height, age at menarche, type-2 diabetes, bone mineral density and gene–diet interactions^48^,^49^,^50^,^51^,^52^. We also observe cases of population-specific effects of DNA methylation variation, such as that of the 6p12.3 enhancer region that was hypomethylated in rainforest hunter-gatherers and under genetic control only in this group (Fig. 3f).

Our analyses identify a gene set showing extensive methylation differences between human groups that started to diverge at least 45,000 years ago—a division corresponding to the second deepest divergence among African populations^7^,^8^,^28^,^30^. In specific cases, we provide a link between DNA methylation variation, genetic diversity and phenotypic traits. For example, the SNP-meQTL detected for *IGF2BP2* (Fig. 3c), as well as those detected at nine other loci, have been directly identified as presenting the strongest association signals for various phenotypes, including height, by GWAS (Supplementary Data 1). In doing so, our study motivates further work to understand the mechanistic links between the patterns of epigenetic variation observed and the extensive phenotypic diversity characterizing African populations.

Third, we show that genetic variants associated with DNA methylation variation are enriched in signals of positive selection. That these signals appear to be more pronounced among agriculturalist populations, both in the western and eastern settings, suggests the occurrence of increased local adaptation targeting regulatory variation in these human groups. One of the most iconic phenotypes distinguishing rainforest hunter-gatherers and farmers is small body size^26^, the genetic and adaptive bases of which are increasingly recognized. Recent studies have reported several independent loci with adaptive alleles that appear to correlate with height, supporting a scenario of convergent evolution related to the African ‘Pygmy' phenotype^5^,^40^,^41^,^42^. Among the candidate loci proposed, the *CISH*–*MAPKAPK3*–*DOCK3* region in chromosome 3 presents both signals of selection and association with height^40^. Specifically, genetic variation at *DOCK3* has been associated with height in Europeans^52^ and, together with *CISH*, which is involved in the human growth hormone pathway, presents a suggestive association in a combined RHG–AGR sample^40^. Furthermore, variants of *CISH* have been associated with susceptibility to infectious disease, including tuberculosis and malaria, in several African populations^53^.

Interestingly, we find that *CISH*, *MAPKAPK3* and *DOCK3* are differentially methylated between populations, owing to meQTLs that show strong population differentiation between rainforest hunter-gatherers and farmers (*F*_ST_=0.17–0.23, with longer branch lengths among RHG, among the 5% highest of the genome). Likewise, the height-associated SNP rs16860216 at *IGF2BP2* (ref. 52), which we also find to control methylation variation, presents strong allele frequency differences between groups (*F*_ST_=0.15, with longer branch length among AGR, among the 5% highest of the genome). Collectively, these results provide new insight into how DNA methylation variation might have participated, through its association with genetic variants, to adaptive phenotypes, including the Pygmy phenotype, broadening our understanding of hunter-gatherer and farmer evolutionary ecology.

In summary, this study substantially increases our understanding of the relative impacts that population genetic variation and differences in lifestyles and ecologies have on the human epigenome, and illustrates the utility of DNA methylation as a marker to track variation in regulatory activity following environmental change. Furthermore, our findings suggest that populations can initially respond to environmental challenges via epigenetic changes, uncoupled from variation in the DNA sequence, with the adaptive phenotype increasingly being achieved via genetic changes as time passes. We thus provide a basis for further experimental and theoretical studies assessing the role of epigenetic mechanisms in human adaptation over different time scales.

## Methods

### Population samples

We studied peripheral whole blood DNA from a total of 381 samples, corresponding to 362 individuals and 19 replicate samples, from seven populations located across the Central African belt (Fig. 1a; Table 1). These populations can be divided into two main groups: RHG populations, historically known as ‘pygmies', who have traditionally relied on the equatorial forest for subsistence and who live close to, or within, the forest; and AGR populations, living either in rural/urban deforested regions or in forested habitats in which they practice slash-and-burn agriculture. The w-RHG sample consisted of 112 Baka from Minvoul (Gabon) and the regions of Oveng-Djoum, Lomié-Messok, and Salapoumbe (Cameroon). Given the highly similar methylation and genetic profiles of the Baka individuals from Cameroon and Gabon (Fig. 1b; Supplementary Fig. 5a,c), and their residence in the same ecological habitat (Table 1), we pooled these samples in a single group. The e-RHG sample consisted of 47 unrelated Batwa from the surroundings of the Bwindi Impenetrable Forest in southwest Uganda, all of whom were born in the forest^42^. The w-AGR sample contained 55 Nzebi from Libreville (Gabon) and 39 Fang from Yaoundé (Cameroon). Again, based on the similarity of their methylation and genetic profiles (Fig. 1b; Supplementary Fig. 5b,c) and habitats (Table 1), these samples were merged into a single group. The e-AGR sample contained 48 Bakiga from the surroundings of the Bwindi Impenetrable Forest in southwest Uganda^42^. We also analysed an AGR sample of 61 Nzime from Messok (Cameroon) (referred to as f-AGR), who were recruited on the basis of their frequent practice of hunting in the forest traditionally inhabited by the w-RHG sample.

Further details about the modes of subsistence of these populations, their habitats and sample sizes, before and after filtering, are provided in Table 1. Informed consent was obtained from all participants and from both parents of any participants under the age of 18. Ethical approval for this study was obtained from the institutional review boards of Institut Pasteur, France (RBM 2008-06 and 2011-54/IRB/3), Makerere University, Uganda (IRB 2009-137) and University of Chicago, USA (16986A).

### Genotyping data

Of the 362 individuals included in this study, 191 had already been genotyped by Illumina Omni1 in two previous studies^10^,^42^. This consisted of 46 w-RHG, 15 e-RHG, 29 w-AGR, 31 e-AGR and 21 f-AGR individuals from ref. 10, and 34 e-RHG and 15 e-AGR individuals from ref. 42. The remaining 171 samples—105 w-RHG, 26 w-AGR and 40 f-AGR individuals—were genome-wide genotyped using the Illumina OmniExpress for 719,665 SNPs. We filtered out 7,120 SNPs on the basis of their physical location (that is, those on the Y-chromosome and SNPs unmapped on dbSNP build 37), problematic genotype clusters in GenomeStudio (Illumina, San Diego) based on a GenTrain score <0.35, and SNP call rate <95%. We also filtered out two w-RHG individuals with a call rate <95% and eight individuals presenting cryptic relatedness (that is, kinship coefficient >0.15 with another individual), with the KING program^54^.

We phased the 191 Omni1 individuals with SHAPEIT2 (ref. 55) and imputed missing SNPs in the OmniExpress data set, using the Omni1 data set as a reference, with IMPUTE2 (ref. 56). Five samples (4 w-RHG and 1 f-AGR) with call rates <95% after imputation were removed. After filtering out low-quality imputed SNPs and SNPs with call rate <95% after imputation, we obtained a final set of genotypes at 876,886 SNPs for 347 individuals, comprising 98 w-RHG, 94 w-AGR, 60 f-AGR, 47 e-RHG and 48 e-AGR individuals. To evaluate imputation accuracy, we compared the concordance of genotyped and imputed SNPs with whole-genome sequences from 20 w-RHG (Baka) and 20 w-AGR (Nzebi) studied here, obtained by Illumina HiSeq 2000 at an average coverage of 5.6 × (17,080,726 SNPs, unpublished data). SNP calling of next-generation sequencing data was performed with GATK^57^. We kept SNPs passing a sensitivity threshold (VQSR tranche) of 99.9%, with a confidently called reference allele, passing Hardy–Weinberg equilibrium and found in genomic regions of ‘strict callability' (as defined by the 1000 Genomes Project Consortium^58^) and limited evidence of identity-by-descent (IBD). Average concordance rate was 97.2% (individual range: 94.6–99.6%) and 96.5% (individual range: 94.2–98.6%) for genotyped and imputed SNPs, respectively. Finally, we had to remove another two individuals because of their methylation profiles (see the ‘DNA methylation data processing' section), yielding a final data set of 345 individuals for whom we had both genotype and methylation data.

### Genome-wide DNA methylation analysis

Genome-wide DNA methylation data at more than 485,000 sites was obtained using an Infinium HumanMethylation450 BeadChip. Bisulfite conversion of 750 ng of genomic DNA was performed with the EZ DNA Methylation Kit. Successful conversion was confirmed by methylation-specific PCR before proceeding with subsequent steps of the Infinium assay protocol. The bisulfite-converted genomic DNA was isothermally amplified at 37 °C for 22 h, enzymatically fragmented, purified and hybridized with the HumanMethylation450 BeadChip at 48 °C for 18 h. Each BeadChip was then washed to remove any unhybridized or non-specifically hybridized DNA. Labelled single-base extension was performed with bead-bound probes hybridized to the DNA, and the hybridized DNA was removed. The extended probes were stained with multiple layers of fluorescence, and the BeadChip was then coated with a proprietary solution and scanned with the Illumina iScan system. Raw data were processed with Genome Studio Methylation Module software.

### Targeted pyrosequencing

Bisulfite PCR-pyrosequencing assays were designed with PyroMark Assay Design 2.0 (Qiagen). The regions of interest (*IGF2BP2* cg23956648, *HOXC6* cg21582112, *ZNF492* cg09314196, 6p12.3 enhancer region cg23053977, *DOCK1* cg06406458, *COL23A1* cg08684511, *RORA* cg09879458, and *ADAM28* cg18757155) were amplified by PCR, using the HotstarTaq DNA polymerase kit (Qiagen) as follows: 15 min at 95 °C (to activate the *Taq* polymerase), 45 cycles of 95 °C for 30 s, 58 °C for 30 s and 72 °C for 30 s, with a final 5-min extension step at 72 °C. For pyrosequencing, a single-stranded DNA was prepared from the PCR product with the Pyromark Vacuum Prep Workstation (Qiagen), and sequencing was performed with sequencing primers on a Pyromark Q96 MD pyrosequencer (Qiagen). Methylation levels were calculated for each CpG dinucleotide with Pyro Q-CpG software (Qiagen). The primer sequences are listed in Supplementary Table 7.

### DNA methylation data processing

In total, 381 samples were hybridized with the HumanMethylation450 array, including 362 unique samples and 19 technical replicates. We removed probes that potentially cross-hybridize^59^, those on the X and Y chromosomes, and those containing SNPs, or associated with CpGs containing SNPs, at a frequency higher than 1% in at least one of the studied populations. The list of SNPs was based on (i) our own genotyping data set for more than 876,886 SNPs genome-wide (see ‘Genotyping data' section), and (ii) the whole-genome sequencing data set for 20 w-AGR and 20 w-RHG individuals mentioned above. Following this filtering process, 365,886 of the original 485,512 sites on the array were retained. We calculated methylation levels from raw data, using the R bioconductor lumi package. The *M*-value has been shown to provide better detection sensitivity than β-values at extreme levels of modification^34^. We therefore used the *M*-value unless otherwise stated. *M*-values were then adjusted for background and colour bias with lumi, and quantile normalized. We corrected for technical differences between Type I and Type II assay designs, by performing subset-quantile within array normalization on *M*-values with the R bioconductor minfi package^60^. PCA showed that a batch effect explained part of the variance (Kruskal–Wallis *P* value of 8.35 × 10^−55^ for PC2) of the normalized data, and we used the ComBat function from the sva bioconductor package to correct for this effect^61^. Two samples (1 w-RHG and 1 f-AGR) were removed because they presented a clear excess of hemi-methylated sites.

### Accounting for age and heterogeneity in cell subtypes

To account for the potential confounding introduced by age and cellular heterogeneity in whole blood, we first estimated these variables in all samples. Ages were estimated from methylation data for all samples, with an elastic net regression model^32^, and the estimated ages were compared with the ages declared, when these were available (Supplementary Note 2). To account for cellular heterogeneity, we used a reference-based method in which the DNA methylation signature of each of the principal types of immune cells (granulocytes, monocytes, B cells, CD4^+^ T cells, CD8^+^ T cells and NK cells) was used to predict the proportions of these cell types in unfractionated whole blood^33^. Predictions for white blood cell types were obtained by applying the ‘estimateCellCounts' function of the minfi package^60^ to the normalized β-values. This function was modified slightly to accept a matrix of β-values rather than an RGSet object. The resulting estimated cell counts were rescaled to 1. We also determined the relative proportions of various cell subtypes (CD4^+^ T cells, CD8^+^ T cells, B cells and NK cells) among the PBMCs of 35 e-RHG and 31 e-AGR subjects, by fluorescence-activated cell sorting (FACS; Supplementary Note 3). Note that the set of probes that were used to predict heterogeneity in blood cell composition^33^ were removed, yielding a final set of 365,401 probes that were used in all the subsequent analyses. Estimated ages and cell subtype heterogeneity across populations were then used to adjust *M*-values for all analyses, including principal component analyses, the estimation of differentially methylated sites and the mapping of meQTLs.

### Determination of differentially methylated sites

Sites differentially methylated between populations (DMS) were identified statistically, by fitting a linear regression model for each site (*M*-values ∼population+sex+age+cell type proportions+error), and applying empirical Bayes smoothing to the s.e.'s, with the R bioconductor limma package^62^. Sites with a Benjamini and Hochberg adjusted *P*<0.01 were considered to be differentially methylated. To define the amplitude of DMS, we used different criteria: a Benjamini and Hochberg adjusted *P* value <0.01 and a difference in mean methylation level between the two populations of more than 2, 5 or 10%. For this analysis, methylation level was determined as the ratio of methylated probe intensity to overall intensity, the β-value^34^. We extracted the overlaps between different DMS sets and calculated the *P* values measuring the probability of these overlaps being obtained by chance, using 10^7^ resamplings. DNA methylation levels at targeted sites are strongly correlated within regions of about 2,000 bp^20^. Thus, for each DMS list, we randomly resampled the same number of sites from all 365,401 sites, taking into account the distance between the DMS.

### Genomic features of differentially methylated sites

We analysed the enrichment in target sites of particular genomic regions, by calculating an OR, defined as follows:


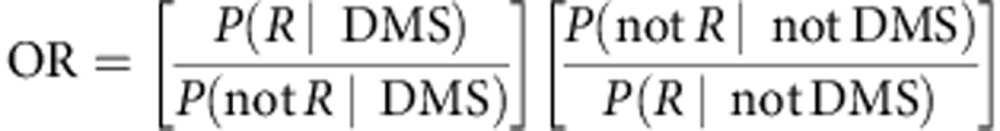


with *R* being ‘in the region'.

Genic regions were defined according to the UCSC_REFGENE_GROUP column from the Illumina HumanMethy450 annotation: distal promoter (from 1,500 to 200 bp upstream from the TSS), proximal promoter (less than 200 bp upstream from the TSS), 5′UTR, first Exon, Gene Body and 3′UTR. Histone modification peak data for H3k4me1, H3K4me3, H3K9me3 and H3K27me3, which correspond to the histone marks for which data was available for PBMCs, were downloaded from the ENCODE website (http://genome.ucsc.edu/ENCODE/). A site was considered to colocalize with a histone modification mark if it falls into the region defined as a ‘narrow peak' (FDR of 0.01). TF-binding sites affinity scores for sequences of 30 bp around each methylation site were obtained using the TRAP software^63^ and the position weight matrix of 85 human TFs from JASPAR^64^. For each TF, a site was considered to have a high affinity if it fell into the top fifth percentile of the score distribution. *P* values for enrichment in genomic positions, histone marks or TF-binding sites among recent and ancient DMS were obtained using a *χ*^2^-test.

### Biological functions of differentially methylated genes

We extracted all differentially methylated genes, defined as genes carrying at least one DMS. We used the goseq R bioconductor package to perform an analysis of the over-representation of gene ontology categories^65^ among differentially methylated genes. We fed the number of probes corresponding to each gene into the probability weighting function of the goseq package. As not all the genes of the genome are represented on the Illumina HumanMethy450 BeadChip, our reference set in the over-representation analysis consisted of the 19,672 genes for which we had data. DMS sets were significantly enriched in a given category if the FDR-adjusted *P* value was <0.05.

### Mapping of meQTLs

We identified meQTLs with a Bayesian statistical framework implemented in the eQtlBma package, which was specifically designed for the detection of QTLs jointly in multiple subgroups^66^. We filtered out SNPs with an allele frequency below 10% in all populations. Age, sex and the proportions of the various cell types were used as covariates in the linear model. In addition, we included the first PC obtained from genotyping data as a covariate, to correct for varying degrees of AGR ancestry across individuals within RHG populations. We then estimated the genome-wide weight of each configuration (Supplementary Table 6) using eqtlbma_hm and the default grids provided by the eQtlBma package as a priori for the hierarchical model. The probability of a methylation site having no meQTL (*π*0) was estimated by the EBF method^67^, and various posterior probabilities were calculated with eqtlbma_avg_bfs. We then extracted all the methylation sites with at least one meQTL at an FDR of 0.01 (ref. 68). We identified the best-associated SNPs, defined as all SNPs for which the sum of posterior probabilities for being the best-associated SNP, assuming that the site was associated with only one SNP, was at least 0.85. For most sites with several SNPs associated with high posterior probabilities, the best configurations (that is, the combination of populations in which the SNP was a meQTL) were identical for all the SNPs. In the 2,469 cases in which there were at least two configurations, the best configuration was chosen by looking directly at the association. The 155 cases for which there were more than two different configurations were discarded from the list of significant meQTL-associated sites.

We calculated the proportion of historical DMS either associated with meQTLs presenting strong differences in allele frequency between the populations compared (that is, high *F*_ST_) or reflecting G × G/G × E interactions (that is, meQTLs that are detected only in some populations) using an analysis of variance (*M*=population+genotype+population × genotype). We thus obtained the proportion of the variance in DNA methylation levels explained by each factor and their corresponding *P* values. After adjustment for multiple testing, using a Benjamini and Hochberg correction, we considered that a meQTL-associated DMS reflected G × G/G × E interactions when *P*<0.01 for the population × genotype factor.

### Detection of positive selection

To detect mutations presenting signals of positive selection, we used the analysis of molecular variance-based *F*_ST_ (ref. 37), the LSBL^38^ and the haplotype-based iHS^39^. For LSBL, we choose the Ju/'hoansi Khoe-San as outgroup, because genetic distances between this population and RHG and AGR groups were similar. We merged our imputed SNP genotyping data set with the HumanOmni2.5 data set of the Khoe-San from Schlebusch and colleagues^7^, and kept 664,661 shared SNPs that presented neither allele mismatches nor allele frequency discordances (determined by comparing w-AGR with south-African Bantu speakers). To measure the enrichment in high *F*_ST_ and LSBL among meQTLs, we compared the proportions of high *F*_ST_ or LSBL values (defined as the 5% highest values genome wide) between meQTLs and all the remaining SNPs located in a 20-kb window centred on each HumanMethylation450K probe. Statistical significance was tested with a Cochran–Mantel–Haenszel test, stratifying data by bin of derived allele frequencies (from 0 to 1, in 0.1 steps). iHS values were computed for our entire set of 876,886 SNPs, and normalized by bin of derived allele frequencies (from 0 to 1, in 0.025 steps) in each of the five populations separately (w-RHG, w-AGR, f-AGR, e-RHG and e-AGR). Ancestral states of the SNPs were determined using the sequence provided by the 1000 Genomes Project^58^. We used a *χ*^2^-test to compare the proportion of high |iHS| values (defined as the 5% highest |iHS| values genome wide) between meQTLs and all the remaining SNPs located in a 20-kb window centred on each HumanMethylation450K probe. We filtered out SNPs with LD *r*^2^ values >0.8 in each pair of populations merged, for *F*_ST_, or in each population separately, for LSBL and iHS, using plink^69^.

### Annotation using data from genome-wide association studies

For all sets of DMS genes and meQTLs, we explored their implication in human diseases and traits using hits of GWAS, obtained from the 02/06/2015 version of the NHGRI database, which we manually modified to include two recent GWAS of height^52^ and age at menarche^51^. Only GWAS signals with *P* values <5 × 10^−8^ were considered. We used two approaches; a gene-based approach and a SNP-based approach. The gene-based approach relies on the simple fact that a DMS gene is the reported gene of a GWAS hit. A set of *n* DMS genes is considered enriched in GWAS genes if the proportion of DMS GWAS genes in this set is larger than in 95% of 10,000 randomly sampled sets of *n* genes. Genes are randomly sampled from all genes that have at least one methylation probe in the HumanMethylation450 BeadChip, and are matched to the observed number of probes per gene observed in the tested set. We also tested if sets of DMS genes were enriched in genes associated to individual diseases/traits. *P* values were obtained by resampling. Only diseases/traits that were associated with more than five DMS genes were considered.

The second SNP-based approach evaluates if meQTLs correspond to, or are in strong linkage disequilibrium (*r*^2^>0.8) with, SNPs reported as best GWAS hits. For each set of meQTLs, we first removed all SNPs in LD using *plink* (‘--indep-pairwise 50 5 0.8')^69^. We next retrieved SNPs in strong linkage disequilibrium with any of these SNPs, using the correlation coefficient implemented in *plink* calculated in our imputed genotyping data set. We then obtained the proportion of GWAS best signals among meQTLs and SNPs in LD with them. To test for enrichments in GWAS hits, we estimated this proportion, using the same procedure, in 10,000 random samples of independent SNPs that were selected to be close to methylation probes.

## Additional information

**Accession codes:** The genotyping data generated in this study have been deposited in the European Genome-Phenome Archive under accession codes EGAS00001000605, EGAS00001000908 and EGAS00001001066. The DNA methylation data generated in this study have been deposited in the European Genome-Phenome Archive under accession code EGAS00001001066.

**How to cite this article:** Fagny, M *et al.* The epigenomic landscape of African rainforest hunter-gatherers and farmers. *Nat. Commun.* 6:10047 doi: 10.1038/ncomms10047 (2015).

## Supplementary Material

Supplementary Information Supplementary Figures 1-13, Supplementary Tables 1-7, Supplementary Notes 1-5 and Supplementary References

Supplementary Data 1 African meQTLs identified in genome-wide association studies (GWAS) as associated with human diseases/traits

## Figures and Tables

**Table 1:** Description of historical modes of subsistence and current habitat of populations in the study.

**Population**	**Sampling location(s)**	**Historical mode of subsistence**	**Language family**	**Current habitat/lifestyle**	N*	***N***†	***N***‡
w-RHG Baka	Lomié-Messok, Salapoumbe, Oveng-Djoum, Southeast Cameroon	Hunter-gatherers	Ubangi	Villages in the equatorial rainforest. Slash-and-burn agriculture, subsistence farming, hunting and gathering in the equatorial forest	78	73	68
w-RHG Baka	Minvoul, Northeast Gabon	Hunter-gatherers	Ubangi	Villages in the equatorial rainforest. Slash-and-burn agriculture, subsistence farming, hunting and gathering in the equatorial forest	34	30	29
e-RHG Batwa	Southwest Uganda	Hunter-gatherers§	N. Bantu||	Villages near the forest. Subsistence farming, hunting and gathering in the equatorial forest before settling	47	47	47
w-AGR Nzebi	Libreville, Gabon	Agriculturalists	N. Bantu	Urban	55	55	55
w-AGR Fang¶	Yaoundé, Cameroon	Agriculturalists	N. Bantu	Urban	39	39	39
e-AGR Bakiga	Southwest Uganda	Agriculturalists	N. Bantu	Villages in rural, deforested areas.Subsistence farming in stable deforested area.	48	48	48
f-AGR Nzime	Lomié-Messok, Southeast Cameroon	Agriculturalists	N. Bantu	Villages in the equatorial rainforest, shared habitat with w-RHG Baka from Cameroon (mostly from the Lomié region). Slash-and-burn agriculture, forest hunting	61	60	59

^*^Sample sizes before normalization and filtering.

^†^Sample sizes, after normalization and filtering, used for methylation analyses.

^‡^Sample sizes, after SNP imputation and filtering for low call rates, used for meQTL mapping.

^§^Although, at present, the Batwa RHG do not live in the forest, they hunted and gathered in the Bwindi Impenetrable Forest in southwest Uganda until it became a national park in 1991. All individuals included in this study were born and raised in the equatorial forest, where they lived in non-permanent camps.

^||^N. Bantu stands for Narrow Bantu.

^¶^This sample corresponds to a composite sample of Bantu-speaking individuals from Yaoundé, mostly belonging to the Fang ethnic group.
