# Supplementary material for: The epigenomic landscape of African rainforest hunter-gatherers and farmers
Source: Nat Commun. 2015 Nov 30;6:10047. doi: 10.1038/ncomms10047 (PMC4674682; doi:10.1038/ncomms10047)
Supplement: Supplementary Information — Supplementary Figures 1-13, Supplementary Tables 1-7, Supplementary Notes 1-5 and Supplementary References [file ncomms10047-s1.pdf]

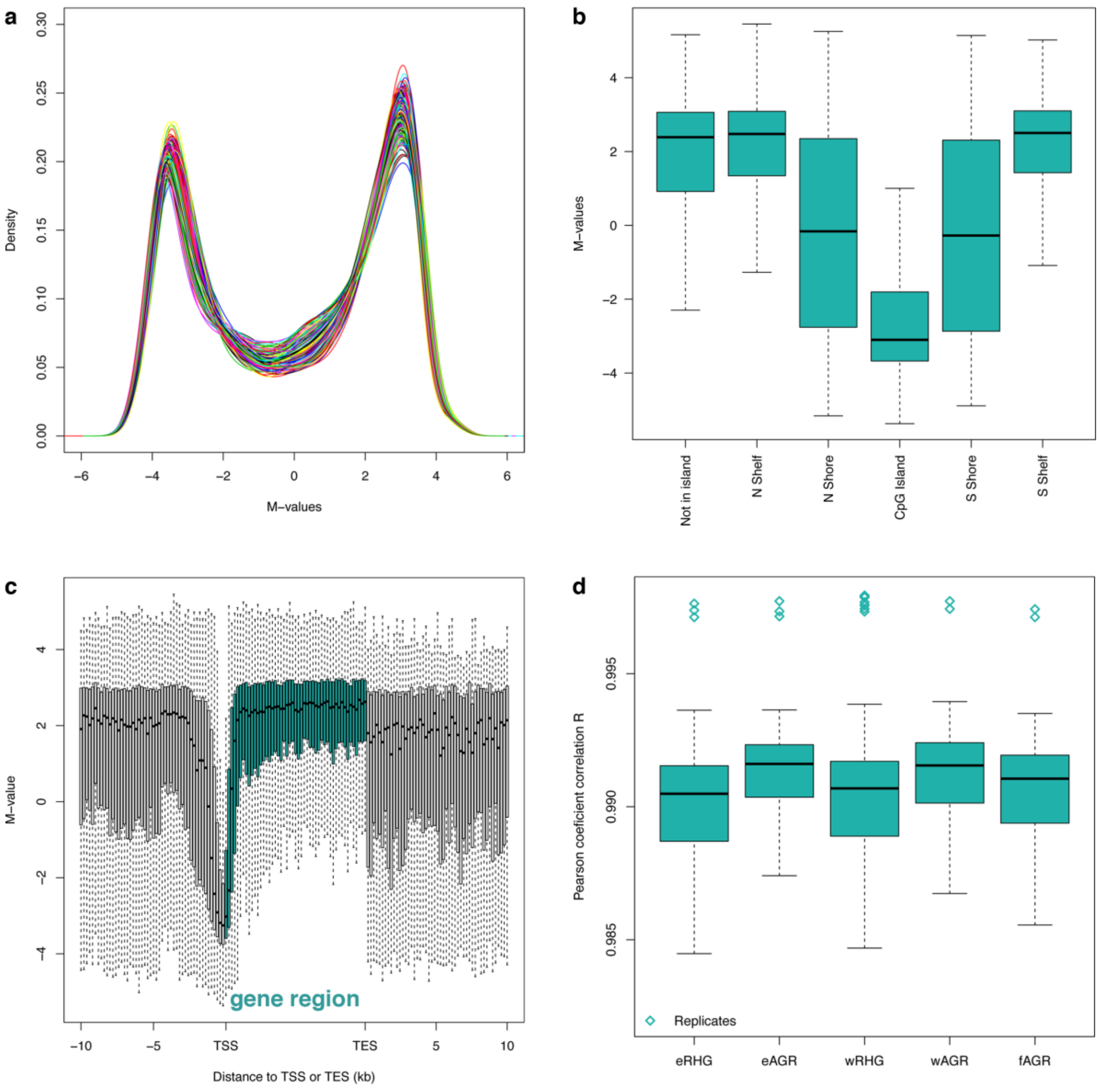

Supplementary Fig. 1. General characteristics of DNA methylation profiles. (**a**) Distribution of methylation M-values after filtering and normalisation. Each line represents the distribution of all M-values for a sample. Negative M-values correspond to unmethylated sites, null M-values correspond to hemi-methylated sites and positive M-values correspond to fully methylated sites. (**b**) CpG island-related distribution of M-values for a representative sample (NZE7003). "Shore" refers to the 0-2kb region from the CpG island on each side; "Shelf" refers to the 2-4kb region from the CpG island on each side; "N" refers to the shore or shelf in 5' of the CpG island. "S" refers to the shore or shelf in 3' of the CpG island. All samples showed the same trends (data not shown). (**c**) Distribution of M-values within 10 kb around gene regions. Sites outside of gene regions were binned in 200bp bins from the TSS or the TES. Gene regions, in blue, were split into 50 equally-sized bins, regardless of the length of the gene. (**d**) Distribution of Pearson's correlation coefficients between methylation M-values of all pairs of samples in each population. Diamonds indicate the very high correlation coefficients between technical replicates (Pearson's $R > 0.997$).

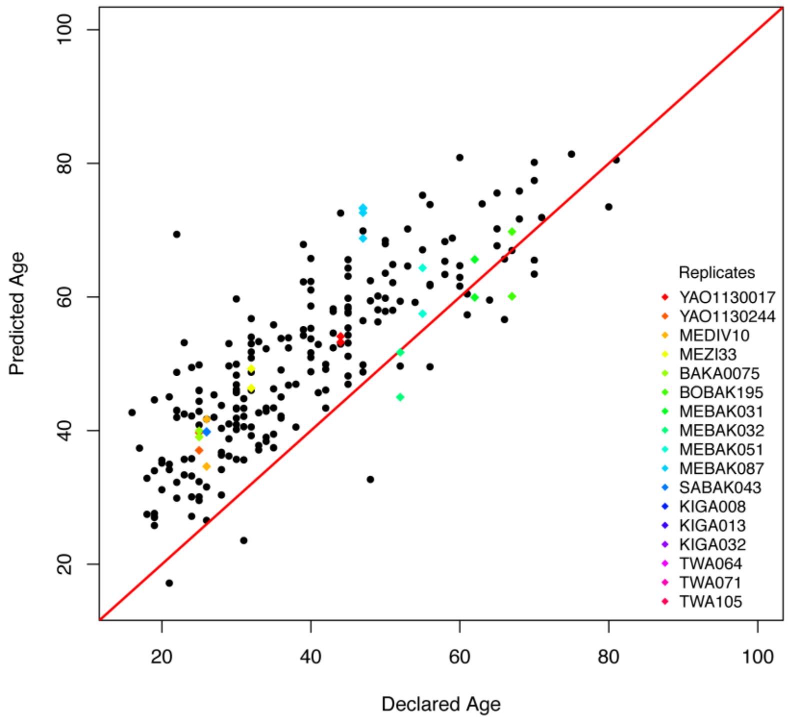

Supplementary Fig. 2. Correlation between declared and predicted ages. Plot showing the correlation between reported ages and predicted ages, the latter being predicted from methylation data for all samples using an elastic net regression model[1]. The colored diamonds represent technical replicates. The slight variance of predicted ages for the same reported age could be due to a general lack of accuracy of the declared age, owing to the absence of systematic registration of people at birth. Although predicted ages were, on average, ~7 years older than declared ages, we observed a high correlation between the two, with a Pearson's correlation coefficient of 0.84 (Supplementary Note 2).

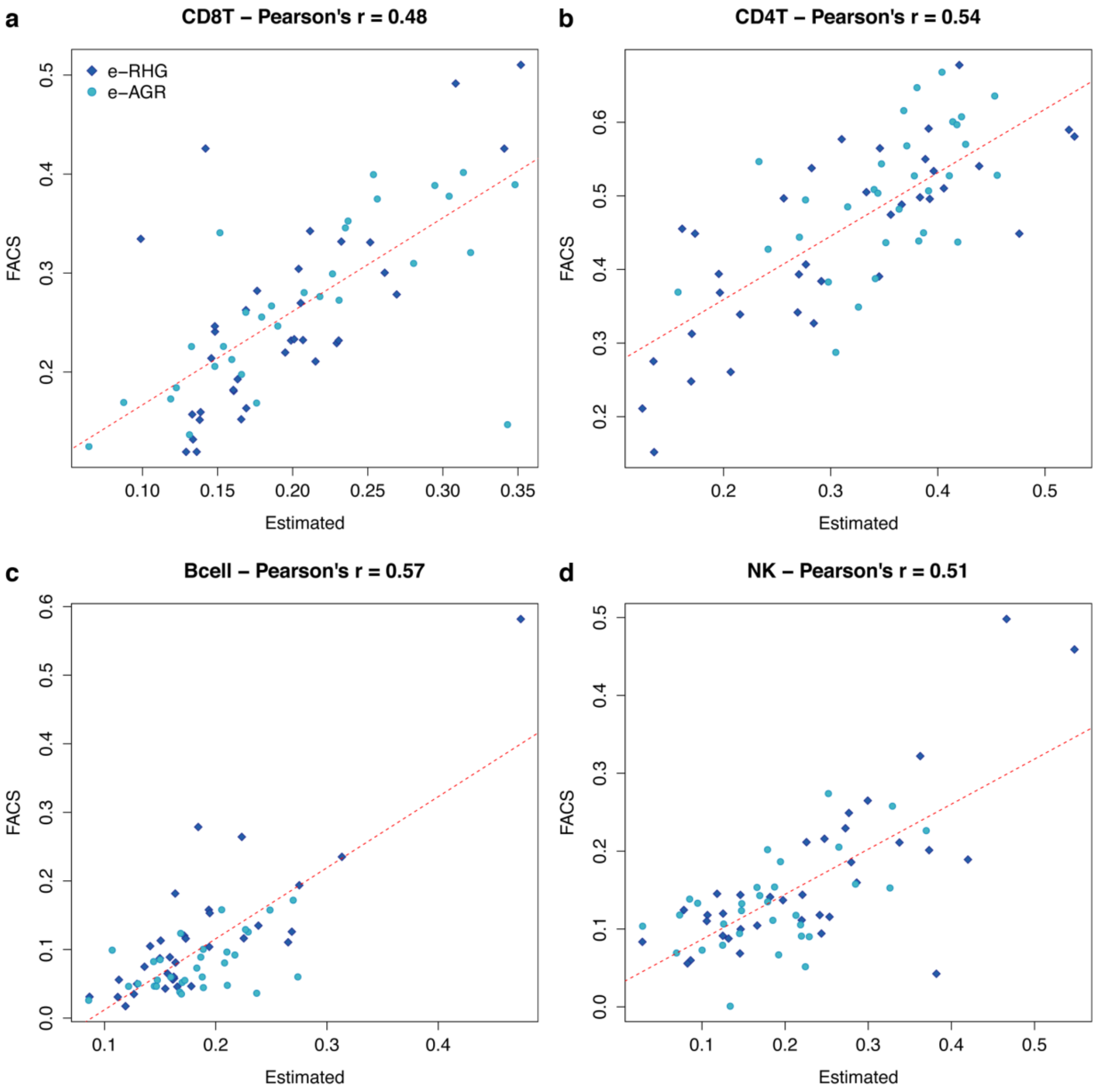

Supplementary Fig. 3. Correlation between predicted and measured proportions of different cell types. Plots showing the correlation between estimated cell proportions based on the DNA methylation signature of each of the principal immune cell components and observed cell proportions, based on FACS data obtained for peripheral blood mononuclear cells (PBMCs) from 35 e-RHG and 31 e-AGR, for (**a**) CD8^{+} T-cells, (**b**) CD4^{+} T-cells, (**c**) B cells and (**d**) NK cells. Dark blue diamonds represent e-RHG samples and light blue circles represent e-AGR samples. Red lines, in each plot, indicate the fitted model of a linear regression for FACS-based ~ estimated cell counts. These results show high correlation coefficients (Pearson's *R*: 0.48-0.57) between estimated and observed proportions (Supplementary Note 3).

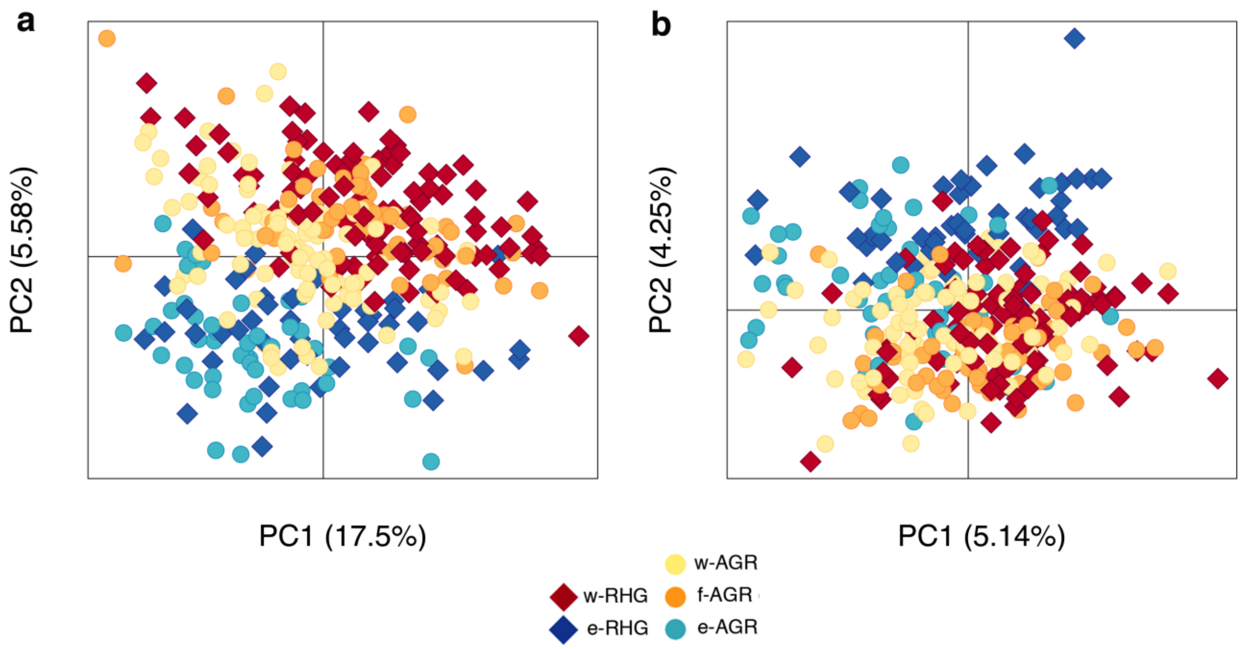

Supplementary Fig. 4. Principal component analyses (PCA) of methylation M-values in all RHG and AGR samples. The proportion of the variance explained by PC1 and PC2 is indicated on the axes. **(a)** PCA of raw, unadjusted M-values using all samples. The first 10 PCs, in particular PC1, strongly correlate with age and variability in cell counts (Supplementary Table 1). (**b**) PCA of gender-, age- and cell counts-adjusted M-values using all samples. In this case, PC1 was highly correlated with RHG/AGR lifestyle ($P = 2.35x10^{-7}$), while PC2 was highly correlated with western/eastern sample location ($P = 3.97x10^{-23}$).

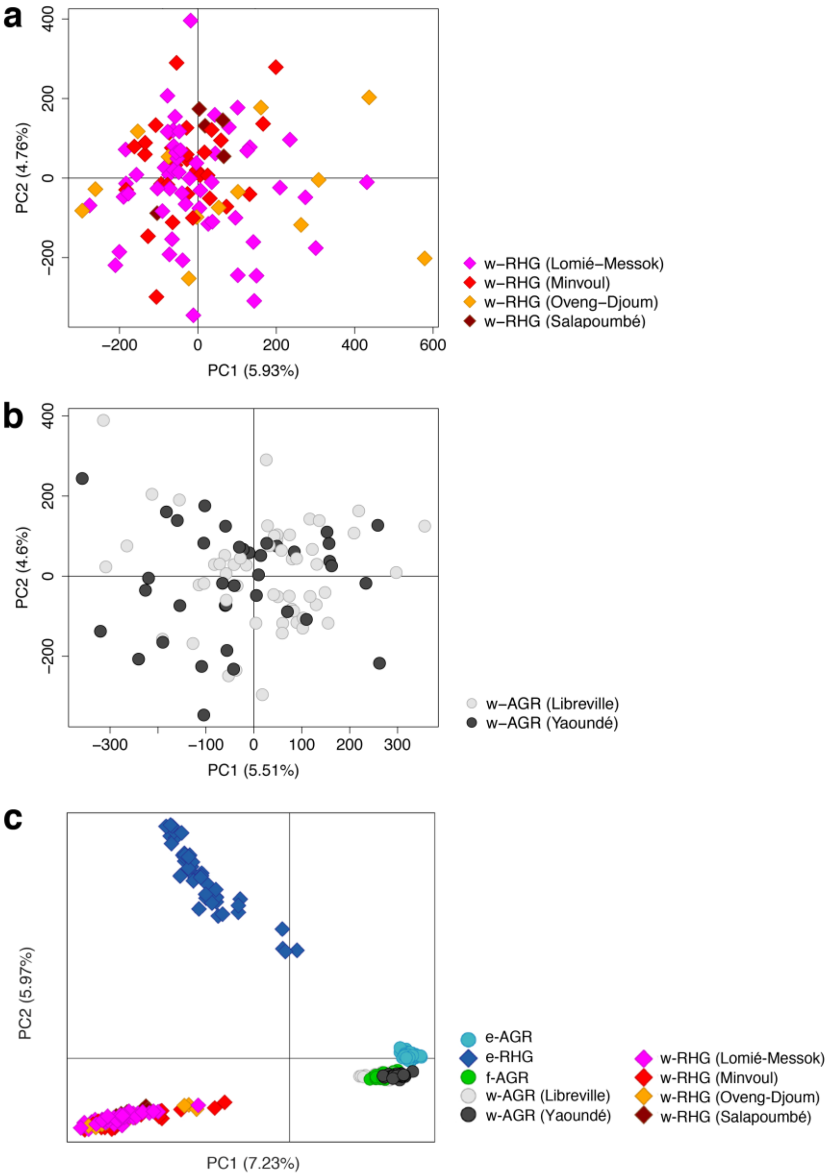

Supplementary Fig. 5. Principal component analysis (PCA) of global DNA methylation and genotyping data in ungrouped populations. (**a**) PCA of genome-wide DNA methylation profiles for the four populations of w-RHG (Table 1). (**b**) PCA of DNA methylation profiles for the two populations of w-AGR, from Libreville and Yaoundé (Table 1). (**c**) PCA of the genotype data for all studied populations, based on 456,507 independent SNPs genome-wide. (**a-c**) The four populations of w-RHG and the two populations of w-AGR are labeled in different colors to show the great similarity in genotype and methylation patterns within w-RHG and w-AGR samples. The proportion of the variance explained by PC1 and PC2 is indicated on the axes. For (**a**) and (**b**) no significant associations were observed between individual population identity and the first two PCs ($P > 0.05$).

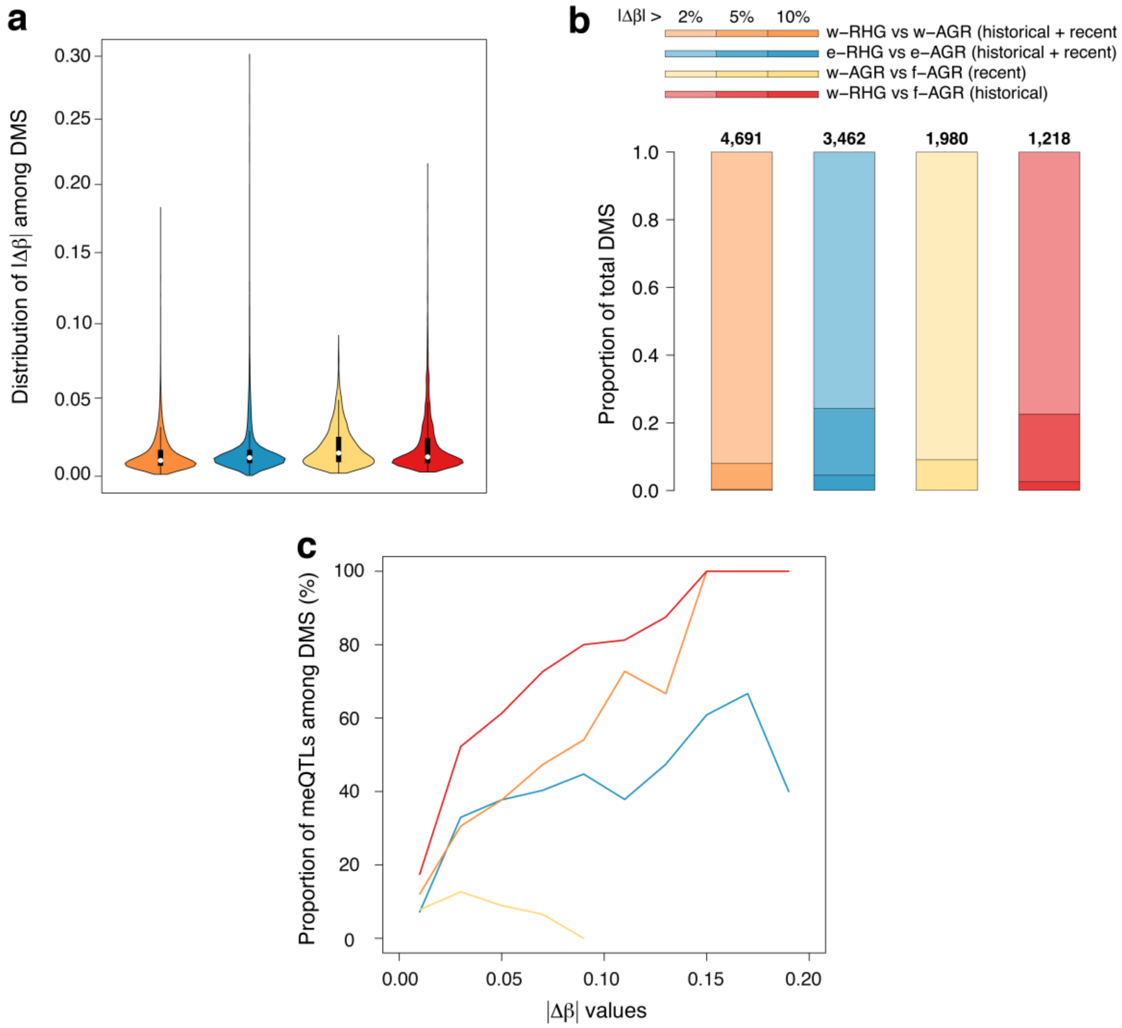

Supplementary Fig. 6. Differences in DNA methylation values between populations. (**a-c**) Colors represent different DMS sets: orange (w-RHG vs w-AGR), blue (e-RHG vs e-AGR), yellow (w-AGR vs f-AGR, *recent* DMS), and red (w-RHG vs f-AGR, *historical* DMS). (**a**) Violin plots representing the distribution of |Δβ| among different DMS. White points represent the median of each distribution. (**b**) Proportions of DMS that present a |Δβ| higher than 5% and 10% among DMS presenting a |Δβ| > 2%, for each population comparison. Numbers on the top of the bars represent the number of DMS in each category. *Historical* DMS presented a significant enrichment in DMS with |Δβ| > 5% with respect to *recent* DMS ($P = 10^{-16}$, χ^2-test). Similarly, *historical* DMS presented 32 DMS with |Δβ| > 10%, whereas *recent* DMS had none. Note that the higher proportion of DMS with |Δβ| > 5% in the e-RHG vs. e-AGR comparison, with respect to the w-RHG vs. w-AGR comparison, may result from (i) some technical differences between the eastern and western sample settings, and/or (ii) the higher F_{ST} at SNPs associated to DMS in the eastern setting (average F_{ST} of 0.06 and 0.04 in the eastern and western settings, respectively) leading to higher |Δβ|. (**c**) Proportion of meQTLs among DMS by bin of |Δβ|. |Δβ| were allocated to 10 bins by steps of 0.02 (the last bin includes all |Δβ| > 0.2). The proportions of DMS associated to meQTLs were then calculated for each bin.

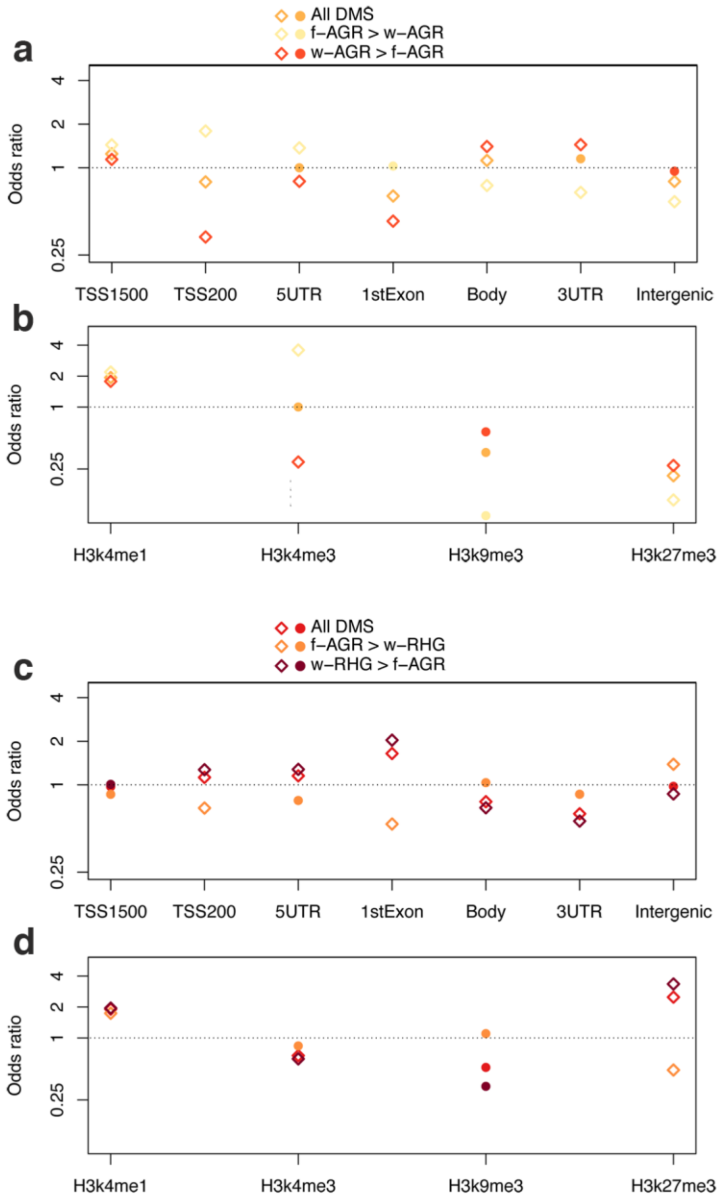

Supplementary Fig. 7. Functional features of DMS. (**a**) Odds ratios measuring the enrichment of different genomic locations in *recent* DMS (5,716 DMS) for all DMS (in orange), DMS more highly methylated in f-AGR with respect to w-AGR (f-AGR > w-AGR, in yellow) and DMS more highly methylated in w-AGR with respect to f-AGR (w-AGR > f-AGR, in red). (**b**) Odds ratios measuring the enrichment of regions mapping to histone modification peaks in *recent* DMS. (**c**) Odds ratios measuring the enrichment of different genomic locations in the set of *historical* DMS (4,049 DMS) for all DMS (in red), DMS more highly methylated in f-AGR with respect to w-RHG (f-AGR > w-RHG, in orange) and DMS more highly methylated in w-RHG with respect to f-AGR (w-RHG > f-AGR, in dark red). (**d**) Odds ratios measuring the enrichment of regions mapping to histone modification peaks in *historical* DMS. In **a-d**, diamonds represent significant odds ratios (P <0.01, χ^2-test).

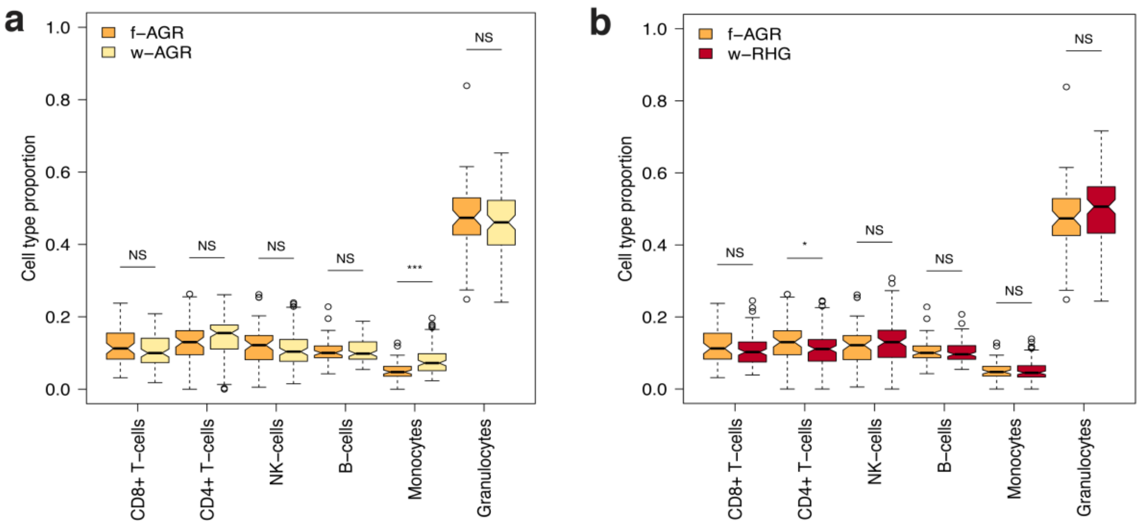

Supplementary Fig. 8. Differences in predicted blood cell counts between populations. Predicted blood cell counts differences for (**a**) the f-AGR/w-AGR population comparison (i.e., used to identify *recent* DMS) and (**b**) the f-AGR/w-RHG population comparison (i.e., used to identify *historical* DMS). No significant differences were detected in any of the population cell counts comparisons, with the exception of an increased number of monocytes in w-AGR (P=2.9x10^{-7}) in the f-AGR/w-AGR comparison, and a slight increased number of CD4^{+} T-cells in f-AGR (P=0.013) in the f-AGR/w-RHG comparison.

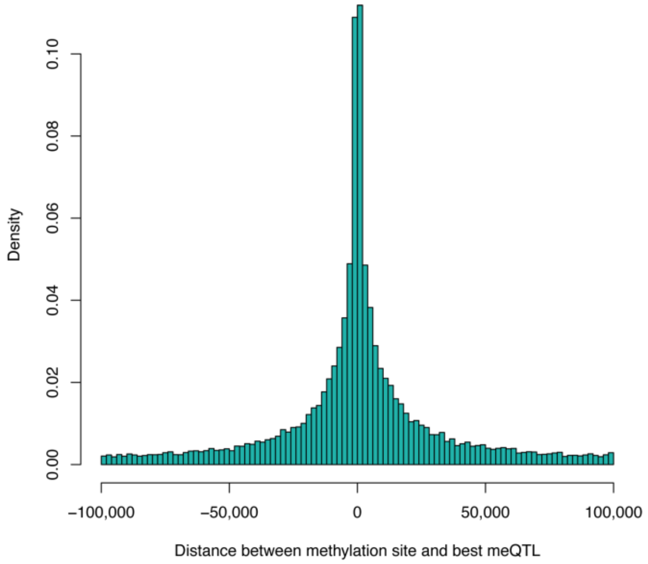

Supplementary Fig. 9. Distance to methylation site of all cis-meQTLs. Histogram representing the density of detected cis-meQTLs as a function of the physical distance from their associated methylation site. Note that 67% of the detected meQTLs were located within 20 kb of the methylation site.

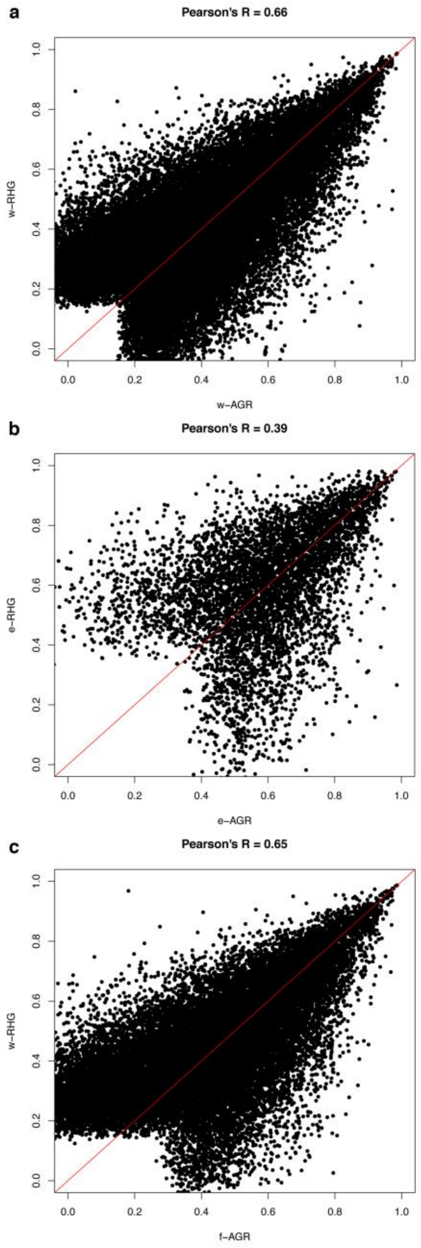

Supplementary Fig. 10. Correlation of meQTL association R^2 between RHG and AGR populations. (**a**) Association R^2 for meQTLs in w-RHG or w-AGR. (**b**) Association R^2 for meQTLs in e-RHG or e-AGR. (**c**) Association R^2 for meQTLs in w-RHG or f-AGR. (**a-c**) For each pair of population, R^2 of associations between SNPs and methylation levels are plotted. meQTLs must be significant at a FDR of 1% either in the RHG or in the AGR population, and with a MAF > 0.1 in both populations. Pearson's correlation coefficients of association R^2 are indicated on the top of each panel.

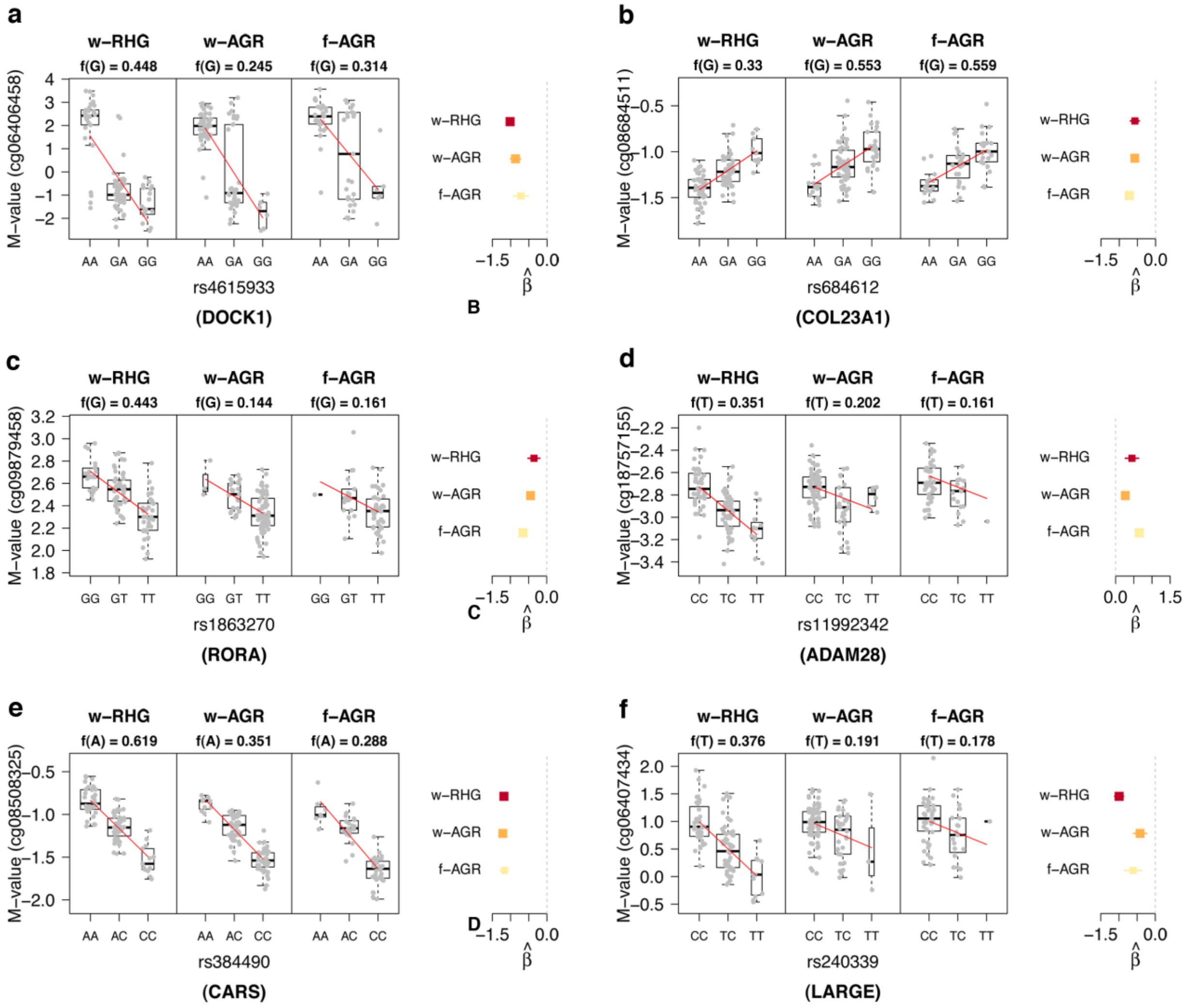

Supplementary Fig. 11. **Genotype-methylation association for a selection of meQTL-DMS.** Examples of meQTLs detected in all populations but presenting different allelic frequencies between RHG and AGR groups. The mean F_{ST} values for the SNP-meQTLs presented here are higher than that observed genome-wide (mean of w-RHG/w-AGR and w-RHG/f-AGR $F_{ST} < 0.03$): (**a**) $F_{ST} = 0.06$ for rs4615933 in *DOCK1*, (**b**) $F_{ST} = 0.09$ for rs684612 in *COL23A1*, (**c**) $F_{ST} = 0.17$ for rs1863270 in *RORA*, (**d**) $F_{ST} = 0.06$ for rs11992342 in *ADAM28*, (**e**) $F_{ST} = 0.16$ for rs384490 in *CARS*, and (**f**) $F_{ST} = 0.08$ for rs240339 in *LARGE*. (**a-f**) For each panel, the three plots on the left represent the distribution of M-values as a function of the genotype, for each population. The minor allele frequency of each meQTL is presented for each population. Red lines indicate the fitted model of a linear regression for M-value ~ genotype for each population. The forest plot on the right of each panel represents the estimated β, i.e., the slope of the linear regression, for each population.

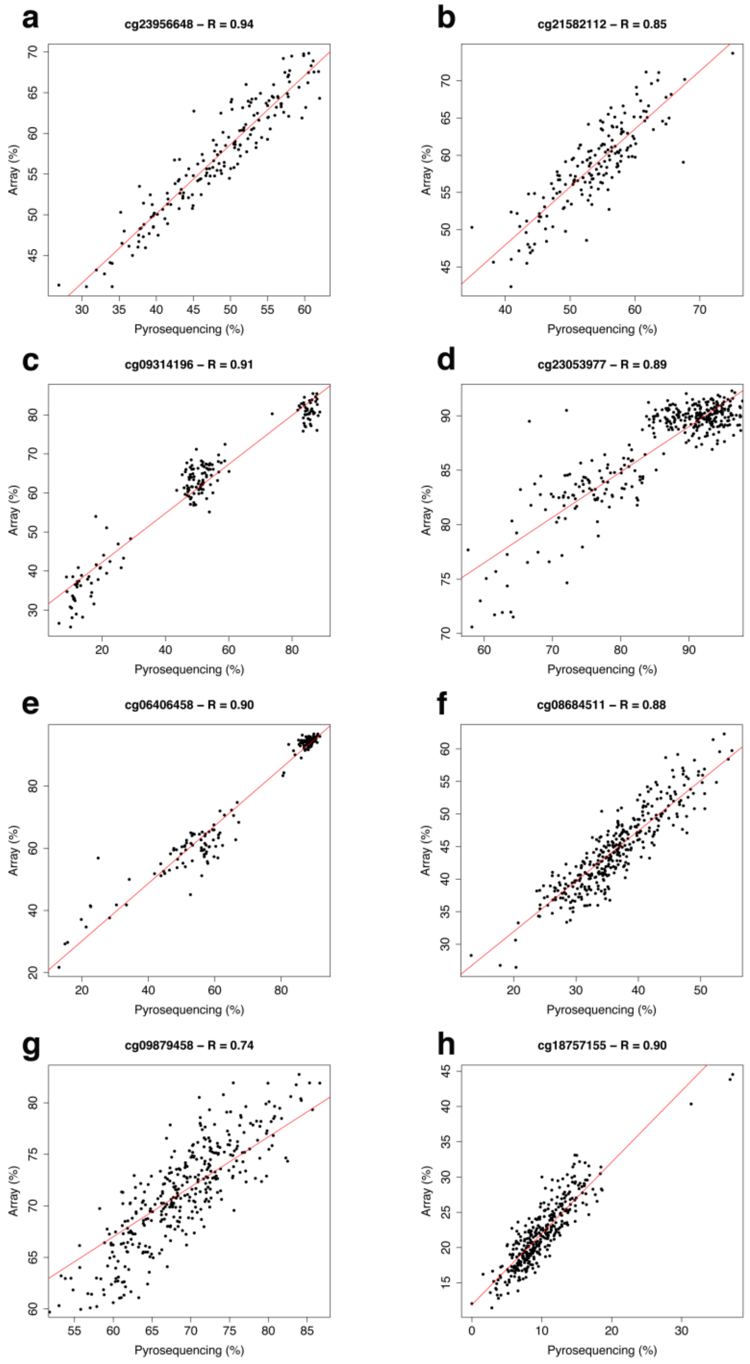

Supplementary Fig. 12. Comparison of DNA methylation profiles obtained by array and pyrosequencing. Red lines correspond to the fit obtained by linear regression (Array ~ Pyrosequencing). Pearson's *R* coefficients are indicated on the top of each panel for (**a**) cg23956648 for *IGF2BP2*, (**b**) cg21582112 for *HOXC6*, (**c**) cg09314196 for *ZNF492*, (**d**) cg23053977 for Enhancer 6p12.3, (**e**) cg06406458 for *DOCK1*, (**f**) cg08684511 for *COL23A1*, (**g**) cg09879458 for *RORA*, and (**h**) cg18757155 for *ADAM28*.

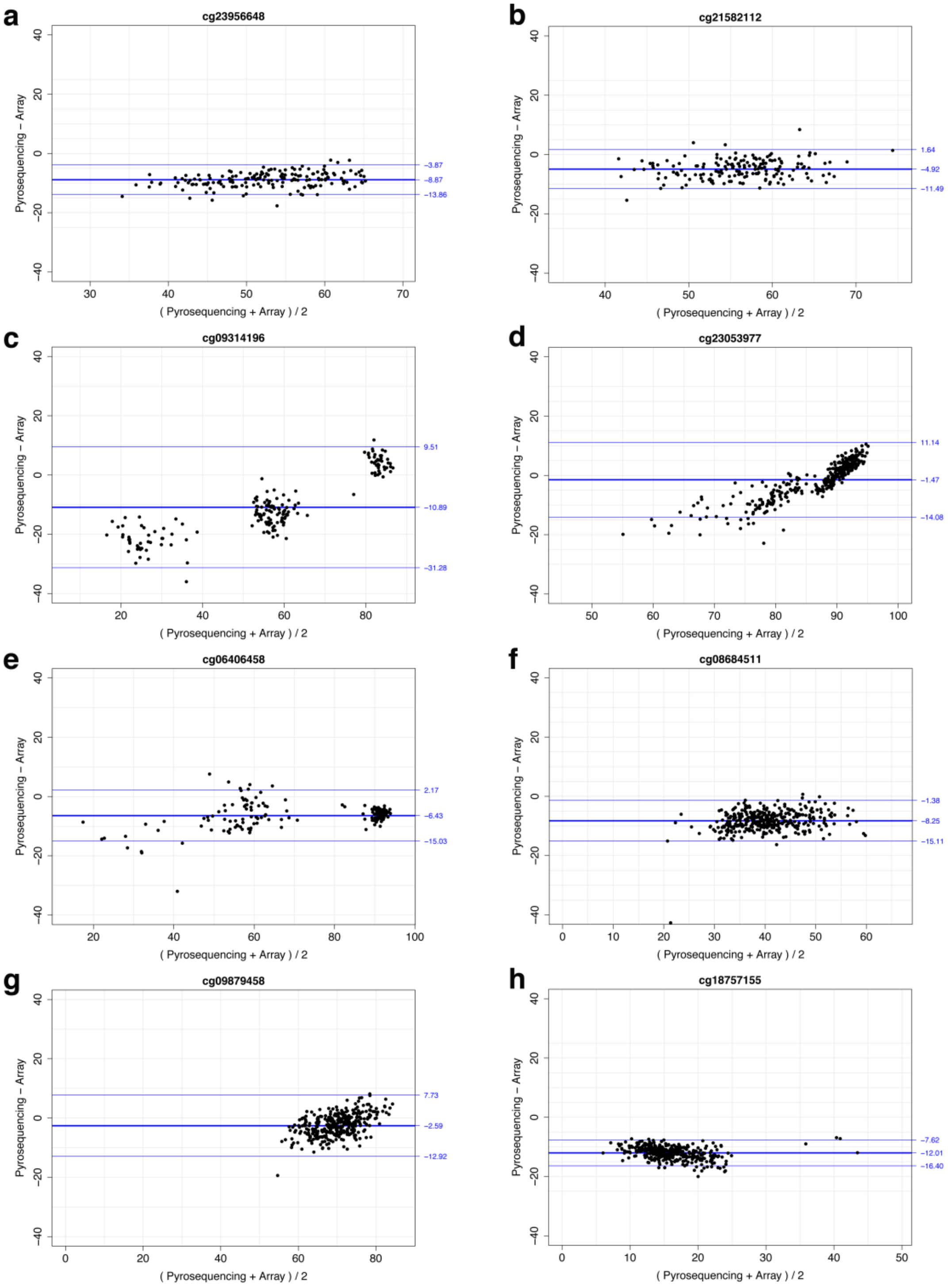

Supplementary Fig. 13. Bland-Altman plots comparing pyrosequencing and array methods. Thick blue lines indicate the mean of Pyrosequencing – Array values. Thin blue lines represent the limits of the 95% confidence intervals for (**a**) cg23956648 for *IGF2BP2*, (**b**) cg21582112 for *HOXC6*, (**c**) cg09314196 for *ZNF492*, (**d**) cg23053977 for Enhancer 6p12.3, (**e**) cg06406458 for *DOCK1*, (**f**) cg08684511 for *COL23A1*, (**g**) cg09879458 for *RORA*, and (**h**) cg18757155 for *ADAM28*.

Supplementary Table 1. *P*-values of association between PCs and confounder factors, before and after adjustment of M-values for age, gender and variability in blood cell proportions. A univariate linear regression model was used for numeric confounders and a Mann-Whitney U test for categorical confounders (Gender, AGR/RHG, West/East). Significant associations are in bold ($P < 0.05$).

**Principal component**	**Variance explained**	**Age**	**Gender**	**CD8+ T cells**	**CD4+ T cells**	**NK cells**	**B cells**	**Mono-cytes**	**Granu-locytes**	**AGR/ RHG**	**West/ East**
Uncorrected PC1	17.50%	**$<10^{-60}$**	**$2.66\text{x}10^{-4}$**	**$<10^{-60}$**	**$9.72\text{x}10^{-4}$**	**$1.73\text{x}10^{-53}$**	**$2.19\text{x}10^{-7}$**	**$3.88\text{x}10^{-7}$**	**$<10^{-60}$**	**$3.08\text{x}10^{-6}$**	**$9.32\text{x}10^{-8}$**
Uncorrected PC2	5.59%	**$6.46\text{x}10^{-9}$**	**$5.30\text{x}10^{-7}$**	**$1.04\text{x}10^{-11}$**	**$4.69\text{x}10^{-36}$**	0.94	**$5.54\text{x}10^{-3}$**	**$7.37\text{x}10^{-6}$**	**$<10^{-60}$**	**$2.32\text{x}10^{-2}$**	**$2.15\text{x}10^{-36}$**
Uncorrected PC3	2.96%	**$1.36\text{x}10^{-3}$**	**$4.00\text{x}10^{-8}$**	**$5.36\text{x}10^{-3}$**	**$7.90\text{x}10^{-5}$**	0.70	**$6.08\text{x}10^{-4}$**	**$2.09\text{x}10^{-3}$**	**$2.43\text{x}10^{-7}$**	**$2.72\text{x}10^{-12}$**	**$1.72\text{x}10^{-17}$**
Uncorrected PC4	2.58%	**$5.39\text{x}10^{-6}$**	**$4.01\text{x}10^{-3}$**	**$9.33\text{x}10^{-7}$**	**$9.75\text{x}10^{-10}$**	0.67	**$5.95\text{x}10^{-7}$**	0.16	**$4.52\text{x}10^{-14}$**	**$9.38\text{x}10^{-10}$**	**$6.07\text{x}10^{-7}$**
Uncorrected PC5	1.95%	**$6.19\text{x}10^{-2}$**	0.35	0.92	0.62	**$1.74\text{x}10^{-6}$**	**$1.04\text{x}10^{-3}$**	0.63	**$1.46\text{x}10^{-2}$**	**$2.09\text{x}10^{-2}$**	**$1.48\text{x}10^{-4}$**
Uncorrected PC6	1.64%	0.12	0.29	0.53	**$2.02\text{x}10^{-3}$**	0.12	**$5.85\text{x}10^{-3}$**	**$3.97\text{x}10^{-5}$**	**$9.17\text{x}10^{-3}$**	0.30	0.12
Uncorrected PC7	1.37%	0.28	0.34	0.78	**$5.89\text{x}10^{-4}$**	**$6.94\text{x}10^{-3}$**	**$4.22\text{x}10^{-7}$**	**$1.15\text{x}10^{-3}$**	0.44	0.11	0.58
Uncorrected PC8	1.35%	**$4.86\text{x}10^{-2}$**	**$1.08\text{x}10^{-5}$**	0.25	**$4.78\text{x}10^{-2}$**	**$1.04\text{x}10^{-2}$**	**$9.30\text{x}10^{-5}$**	**$2.95\text{x}10^{-4}$**	0.50	0.07	**$2.88\text{x}10^{-3}$**
Uncorrected PC9	1.18%	**$6.57\text{x}10^{-5}$**	**$7.28\text{x}10^{-4}$**	**$2.17\text{x}10^{-2}$**	**$7.20\text{x}10^{-4}$**	**$6.56\text{x}10^{-7}$**	**$<10^{-60}$**	**$1.07\text{x}10^{-6}$**	**$2.48\text{x}10^{-3}$**	0.08	0.05
Uncorrected PC10	0.99%	0.10	0.67	**$4.04\text{x}10^{-2}$**	0.57	0.39	**$2.84\text{x}10^{-4}$**	0.52	0.90	0.54	0.44
Corrected PC1	5.14%	1.0	0.87	0.69	0.56	0.50	0.99	0.88	0.42	**$2.35\text{x}10^{-7}$**	**$1.45\text{x}10^{-5}$**
Corrected PC2	4.25%	1.0	0.94	0.77	0.99	0.97	0.08	0.75	0.81	**$1.44\text{x}10^{-6}$**	**$3.97\text{x}10^{-23}$**
Corrected PC3	3.53%	1.0	0.59	0.61	0.72	0.89	0.45	0.91	0.45	0.35	**$1.22\text{x}10^{-3}$**
Corrected PC4	2.63%	1.0	0.72	0.67	0.94	0.99	0.30	0.98	0.88	0.71	**$3.11\text{x}10^{-8}$**
Corrected PC5	2.10%	1.0	0.75	0.86	0.77	0.60	0.71	0.97	0.77	**$1.77\text{x}10^{-2}$**	0.58
Corrected PC6	1.64%	1.0	0.71	0.99	0.99	0.67	0.63	0.38	0.80	0.06	0.36
Corrected PC7	1.57%	1.0	0.55	0.65	0.96	0.92	0.87	0.81	0.37	**$2.36\text{x}10^{-2}$**	**$6.22\text{x}10^{-4}$**
Corrected PC8	1.23%	1.0	0.59	0.30	0.70	0.20	0.58	0.94	0.45	0.86	0.13
Corrected PC9	0.88%	1.0	0.91	0.60	0.60	0.80	0.75	0.98	0.79	**$6.08\text{x}10^{-4}$**	0.13
Corrected PC10	0.87%	1.0	0.55	0.96	0.81	0.49	0.97	0.81	0.96	0.85	0.61

Supplementary Table 2. Enrichment analysis of *historical* and *recent* DMS in TFBS

	TF ID	*P*-value*	OR[§]	SE[†]	Biological functions[¶]
Historical	TFAP2A	$1.95x10^{-6}$	1.42	0.062	kidney, eye, face, body wall, limb and neural tube development; early morphogenesis of the lens vesicle
	NHLH1	$1.55x10^{-3}$	1.32	0.064	cell differentiation; central nervous system development
Recent	GATA3	$8.56x10^{-51}$	2.00	0.046	T-helper 2 (Th2) differentiation; immune response; inflammatory response
	CEBPA	$5.06x10^{-28}$	1.73	0.049	granulocytes, hepatocytes, adipocytes, and cells of the lung and the placenta differentiation
	NFIL3	$5.93x10^{-22}$	1.64	0.050	Activator of interleukin-3 in T-cells; protection of pro-B-cells from apoptosis; regulation of circadian rhythm
	FOXL1	$7.84x10^{-20}$	1.61	0.050	gastrointestinal epithelium cell proliferation and differentiation
	GATA2	$2.09x10^{-19}$	1.60	0.050	endocrine cell lineages differentiation
	HNF1B	$4.20x10^{-17}$	1.57	0.051	embryonic pancreas development; nephron development
	HLF	$1.85x10^{-15}$	1.54	0.051	-
	NKX3-1	$2.05x10^{-14}$	1.52	0.051	prostate development; repressor of glandular epithelium cell proliferation in prostate
	PBX1	$6.93x10^{-13}$	1.49	0.052	sexual development and differentiation; spleen development
	SRY	$3.23x10^{-12}$	1.48	0.052	male development; male sex determination
	SOX9	$5.02x10^{-12}$	1.48	0.052	skeletal development; chondrocyte differentiation
	FOXF2	$7.93x10^{-12}$	1.47	0.052	lung and placenta development
	FOXO3	$5.13x10^{-11}$	1.46	0.052	apoptosis; response to oxidative stress
	FOXA1	$1.65x10^{-9}$	1.42	0.053	embryonic development; liver, pancreas, lung and prostate development; regulation of apoptosis; cell cycle regulation; glucose homeostasis
	HNF1A	$8.51x10^{-9}$	1.41	0.053	gene expression in pancreatic islet cells and in liver; glucose metabolism
	FOXI1	$1.27x10^{-8}$	1.40	0.053	inner ear and kidney development
	ARID3A	$4.14x10^{-8}$	1.39	0.053	control of cell cycle progression; B-cell differentiation
	HOXA5	$2.74x10^{-7}$	1.37	0.053	anterior-posterior axis determination; regulation of development
	MEF2A	$3.95x10^{-7}$	1.37	0.054	activation of growth factor-induced genes; activation of stress-induced genes; skeletal and cardiac muscle development; neuronal differentiation and survival; cell growth and survival; apoptosis

IRF1	$8.40x10^{-7}$	1.36	0.054	regulation of interferon and interferon-inducible genes; host response to viral and bacterial infections; inflammatory response; immune response; hematopoiesis; NK-cells and T-cells differentiation; regulation of cell cycle; apoptosis
RORA	$1.73x10^{-6}$	1.35	0.054	embryonic development; cellular differentiation; T-cells differentiation; immunity; circadian rhythm; lipid, steroid, xenobiotics and glucose metabolism
TBP	$2.44x10^{-6}$	1.35	0.054	Initiation of transcription
FOXD1	$3.48x10^{-6}$	1.34	0.054	retina development; regionalization of the optic chiasm; morphogenesis of the kidney
FOXC1	$4.76x10^{-6}$	1.34	0.054	cell viability; resistance to oxidative stress in the eye
IRF2	$6.86x10^{-6}$	1.34	0.054	repressor of type I IFN and IFN-inducible MHC class I genes; activator of H4 and IL7; cell cycle regulation
CREB1	$3.51x10^{-5}$	1.32	0.054	synchronization of circadian rhythmicity; adipocytes differentiation
MAX	$3.57x10^{-5}$	1.32	0.054	Transcription regulator
T	$6.20x10^{-5}$	1.31	0.055	mesoderm formation and differentiation
NR3C1	$6.73x10^{-5}$	1.31	0.055	inflammatory response; cellular proliferation and differentiation; adipogenesis
SPI1	$9.82x10^{-4}$	1.27	0.055	lymphoid-specific enhancer; macrophages and B-cells differentiation and activation
RUNX1	$1.40x10^{-3}$	1.27	0.055	hematopoiesis; T-cell gene expression regulation
SRF	$1.70x10^{-3}$	1.27	0.055	cardiac cell differentiation and maturation
TAL1::TCF3	$2.25x10^{-3}$	1.26	0.055	hematopoietic differentiation
NR1H2::RXRA	$4.90x10^{-3}$	1.25	0.056	Regulation of cholesterol homeostasis; inhibition of proinflammatory gene expression in atherosclerosis; activation of innate immunity
SOX10	$5.94x10^{-3}$	1.25	0.056	neural crest and peripheral nervous system development

**P*-values were obtained using a chi-squared test to test for enrichment among DMS of high affinity sequences for each transcription factor binding site; [†]Standard errors were obtained using a logistic regression; [§]Odds Ratio measuring the enrichment in high affinity sequences among DMS; [¶]Biological functions were determined using the GeneCards database (http://www.genecards.org)

Supplementary Table 3. Over-representation analyses of Gene Ontology categories among *recent* and *historical* differentially methylated genes using the entire, unfiltered dataset (total of 3,550 genes and 2,128 genes, respectively). Values in bold correspond to FDR-adjusted significant *P*-values.

**Category Name**	**Accession N°**	**FDR-Adjusted *P***	
		Recent	***Historical***
Ontology biological process			
immune system process	GO:0002376	**$8.34x10^{-7}$**	1
immune response	GO:0006955	**$7.26x10^{-6}$**	1
cellular protein metabolic process	GO:0044267	**$1.37x10^{-4}$**	1
interspecies interaction between organisms	GO:0044419	**$1.43x10^{-4}$**	1
symbiosis, encompassing mutualism through parasitism	GO:0044403	**$1.43x10^{-4}$**	1
intracellular transport	GO:0046907	**$1.68x10^{-4}$**	1
multi-organism cellular process	GO:0044764	**$1.86x10^{-4}$**	1
viral process	GO:0016032	**$2.26x10^{-4}$**	1
positive regulation of immune response	GO:0050778	**$3.77x10^{-4}$**	1
protein metabolic process	GO:0019538	**$1.12x10^{-3}$**	1
activation of immune response	GO:0002253	**$1.13x10^{-3}$**	1
single-organism intracellular transport	GO:1902582	**$1.16x10^{-3}$**	1
positive regulation of immune system process	GO:0002684	**$1.35x10^{-3}$**	1
immune response-activating signal transduction	GO:0002757	**$1.85x10^{-3}$**	1
protein transport	GO:0015031	**$3.31x10^{-3}$**	1
regulation of immune response	GO:0050776	**$3.31x10^{-3}$**	1
mRNA metabolic process	GO:0016071	**$3.72x10^{-3}$**	1
organelle organization	GO:0006996	**$3.75x10^{-3}$**	1
establishment of localization in cell	GO:0051649	**$4.02x10^{-3}$**	1
cellular localization	GO:0051641	**$4.76x10^{-3}$**	1
macromolecule localization	GO:0033036	**$4.76x10^{-3}$**	1
immune response-activating cell surface receptor signaling pathway	GO:0002429	**$4.99x10^{-3}$**	1
establishment of protein localization	GO:0045184	**$5.45x10^{-3}$**	1
cell activation	GO:0001775	**$8.46x10^{-3}$**	1
antigen receptor-mediated signaling pathway	GO:0050851	**$8.47x10^{-3}$**	1
cytokine production	GO:0001816	**$9.15x10^{-3}$**	1
defense response to other organism	GO:0098542	**$1.12x10^{-2}$**	1
response to stress	GO:0006950	**$1.12x10^{-2}$**	1
single-organism organelle organization	GO:1902589	**$1.12x10^{-2}$**	1
intracellular protein transport	GO:0006886	**$1.14x10^{-2}$**	1
immune effector process	GO:0002252	**$1.18x10^{-2}$**	1
intracellular signal transduction	GO:0035556	**$1.18x10^{-2}$**	1
regulation of immune system process	GO:0002682	**$1.33x10^{-2}$**	1
cytoplasmic transport	GO:0016482	**$1.38x10^{-2}$**	1
response to wounding	GO:0009611	**$1.63x10^{-2}$**	0.85
T cell receptor signaling pathway	GO:0050852	**$1.63x10^{-2}$**	1
regulation of response to stimulus	GO:0048583	**$1.83x10^{-2}$**	0.84
myeloid leukocyte activation	GO:0002274	**$1.83x10^{-2}$**	1
protein localization	GO:0008104	**$1.83x10^{-2}$**	1
organic substance transport	GO:0071702	**$2.08x10^{-2}$**	1
primary metabolic process	GO:0044238	**$2.08x10^{-2}$**	1

defense response	GO:0006952	**$2.10x10^{-2}$**	1
metabolic process	GO:0008152	**$2.31x10^{-2}$**	1
macromolecule modification	GO:0043412	**$2.45x10^{-2}$**	1
nuclear-transcribed mRNA catabolic process	GO:0000956	**$2.78x10^{-2}$**	1
wound healing	GO:0042060	**$2.84x10^{-2}$**	1
vesicle-mediated transport	GO:0016192	**$2.92x10^{-2}$**	1
translational termination	GO:0006415	**$2.92x10^{-2}$**	NA
cellular protein modification process	GO:0006464	**$3.19x10^{-2}$**	1
protein modification process	GO:0036211	**$3.19x10^{-2}$**	1
leukocyte activation	GO:0045321	**$3.30x10^{-2}$**	1
protein secretion	GO:0009306	**$3.31x10^{-2}$**	1
mRNA catabolic process	GO:0006402	**$3.50x10^{-2}$**	1
RNA catabolic process	GO:0006401	**$3.61x10^{-2}$**	1
lamellipodium assembly	GO:0030032	**$3.83x10^{-2}$**	NA
cellular metabolic process	GO:0044237	**$3.84x10^{-2}$**	1
regulation of T cell activation	GO:0050863	**$4.12x10^{-2}$**	1
response to external biotic stimulus	GO:0043207	**$4.12x10^{-2}$**	1
response to other organism	GO:0051707	**$4.12x10^{-2}$**	1
response to biotic stimulus	GO:0009607	**$4.31x10^{-2}$**	1
nucleobase-containing compound catabolic process	GO:0034655	**$4.32x10^{-2}$**	1
single-multicellular organism process	GO:0044707	1	**$4.36x10^{-4}$**
developmental process	GO:0032502	1	**$7.76x10^{-4}$**
multicellular organismal development	GO:0007275	1	**$7.76x10^{-4}$**
multicellular organismal process	GO:0032501	1	**$7.76x10^{-4}$**
cell fate commitment	GO:0045165	1	**$1.23x10^{-3}$**
single-organism developmental process	GO:0044767	1	**$2.60x10^{-3}$**
nervous system development	GO:0007399	1	**$8.27x10^{-3}$**
central nervous system development	GO:0007417	1	**$9.14x10^{-3}$**
system development	GO:0048731	1	**$9.14x10^{-3}$**
anatomical structure development	GO:0048856	1	**$9.14x10^{-3}$**
cell-cell signaling	GO:0007267	1	**$9.53x10^{-3}$**
signaling	GO:0023052	1	**$1.73x10^{-2}$**
single organism signaling	GO:0044700	1	**$1.73x10^{-2}$**
synaptic transmission	GO:0007268	1	**$1.73x10^{-2}$**
cell communication	GO:0007154	1	**$2.60x10^{-2}$**
tissue development	GO:0009888	1	**$2.60x10^{-2}$**
organ development	GO:0048513	1	**$3.11x10^{-2}$**
neuron fate commitment	GO:0048663	1	**$3.92x10^{-2}$**
organ morphogenesis	GO:0009887	1	**$4.27x10^{-2}$**
cell differentiation	GO:0030154	1	**$4.49x10^{-2}$**
generation of neurons	GO:0048699	1	**$4.49x10^{-2}$**
neurogenesis	GO:0022008	1	**$4.49x10^{-2}$**
neuron differentiation	GO:0030182	1	**$4.49x10^{-2}$**
Ontology molecular function			
protein binding	GO:0005515	**$2.26x10^{-6}$**	1
RNA binding	GO:0003723	**$9.37x10^{-5}$**	1
small molecule binding	GO:0036094	**$5.74x10^{-4}$**	1
poly(A) RNA binding	GO:0044822	**$8.03x10^{-4}$**	1
nucleotide binding	GO:0000166	**$9.73x10^{-4}$**	1
nucleoside phosphate binding	GO:1901265	**$9.76x10^{-4}$**	1
anion binding	GO:0043168	**$2.05x10^{-3}$**	1

ribonucleotide binding	GO:0032553	**$3.72x10^{-3}$**	1
nucleoside binding	GO:0001882	**$4.74x10^{-3}$**	1
purine ribonucleotide binding	GO:0032555	**$5.45x10^{-3}$**	1
ribonucleoside binding	GO:0032549	**$5.45x10^{-3}$**	1
purine ribonucleoside binding	GO:0032550	**$5.60x10^{-3}$**	1
purine ribonucleoside triphosphate binding	GO:0035639	**$5.95x10^{-3}$**	1
purine nucleoside binding	GO:0001883	**$6.02x10^{-3}$**	1
transferase activity	GO:0016740	**$6.88x10^{-3}$**	1
purine nucleotide binding	GO:0017076	**$7.06x10^{-3}$**	1
carbohydrate derivative binding	GO:0097367	**$1.26x10^{-2}$**	1
catalytic activity	GO:0003824	**$1.30x10^{-2}$**	1
heterocyclic compound binding	GO:1901363	**$1.59x10^{-2}$**	1
organic cyclic compound binding	GO:0097159	**$1.62x10^{-2}$**	1
growth factor binding	GO:0019838	0.90	**$4.49x10^{-2}$**
sequence-specific DNA binding	GO:0043565	1	**$9.14x10^{-3}$**
Ontology cellular component			
cytoplasm	GO:0005737	**$7.92x10^{-13}$**	1
intracellular	GO:0005622	**$1.89x10^{-11}$**	1
intracellular part	GO:0044424	**$1.89x10^{-11}$**	1
intracellular organelle part	GO:0044446	**$5.21x10^{-11}$**	1
organelle part	GO:0044422	**$5.21x10^{-11}$**	1
intracellular organelle	GO:0043229	**$6.36x10^{-11}$**	1
cytoplasmic part	GO:0044444	**$3.56x10^{-10}$**	1
organelle	GO:0043226	**$1.98x10^{-9}$**	1
intracellular membrane-bounded organelle	GO:0043231	**$6.58x10^{-9}$**	1
membrane-bounded organelle	GO:0043227	**$2.07x10^{-7}$**	1
cytosol	GO:0005829	**$5.39x10^{-5}$**	1
intracellular non-membrane-bounded organelle	GO:0043232	**$9.66x10^{-5}$**	1
non-membrane-bounded organelle	GO:0043228	**$9.66x10^{-5}$**	1
nucleoplasm	GO:0005654	**$6.33x10^{-4}$**	1
cell-substrate junction	GO:0030055	**$7.11x10^{-4}$**	1
focal adhesion	GO:0005925	**$7.63x10^{-4}$**	1
macromolecular complex	GO:0032991	**$8.03x10^{-4}$**	1
nucleus	GO:0005634	**$8.03x10^{-4}$**	1
nucleoplasm part	GO:0044451	**$1.01x10^{-3}$**	1
cell-substrate adherens junction	GO:0005924	**$1.06x10^{-3}$**	1
nuclear lumen	GO:0031981	**$2.00x10^{-3}$**	1
cell part	GO:0044464	**$2.01x10^{-3}$**	1
cell	GO:0005623	**$2.18x10^{-3}$**	1
intracellular organelle lumen	GO:0070013	**$2.88x10^{-3}$**	1
membrane-enclosed lumen	GO:0031974	**$3.31x10^{-3}$**	1
organelle lumen	GO:0043233	**$3.31x10^{-3}$**	1
nuclear part	GO:0044428	**$4.70x10^{-3}$**	1
organelle membrane	GO:0031090	**$8.47x10^{-3}$**	1
vacuole	GO:0005773	**$9.15x10^{-3}$**	1
vesicle	GO:0031982	**$9.93x10^{-3}$**	1
external side of plasma membrane	GO:0009897	**$1.43x10^{-2}$**	1
ruffle	GO:0001726	**$1.53x10^{-2}$**	1
extracellular membrane-bounded organelle	GO:0065010	**$1.54x10^{-2}$**	1
extracellular organelle	GO:0043230	**$1.54x10^{-2}$**	1
extracellular vesicular exosome	GO:0070062	**$1.54x10^{-2}$**	1

ribonucleoprotein complex	GO:0030529	**$2.28x10^{-2}$**	1
membrane-bounded vesicle	GO:0031988	**$2.31x10^{-2}$**	1
lysosome	GO:0005764	**$2.38x10^{-2}$**	1
lytic vacuole	GO:0000323	**$2.38x10^{-2}$**	1
adherens junction	GO:0005912	**$3.20x10^{-2}$**	1
coated membrane	GO:0048475	**$4.12x10^{-2}$**	1
membrane coat	GO:0030117	**$4.12x10^{-2}$**	1
bounding membrane of organelle	GO:0098588	**$4.13x10^{-2}$**	1
endosome	GO:0005768	**$4.22x10^{-2}$**	1
anchoring junction	GO:0070161	**$4.52x10^{-2}$**	1
integral component of plasma membrane	GO:0005887	1	**$1.02x10^{-3}$**
intrinsic component of plasma membrane	GO:0031226	1	**$6.94x10^{-3}$**
plasma membrane part	GO:0044459	1	**$9.14x10^{-3}$**
integral component of membrane	GO:0016021	1	**$1.73x10^{-2}$**
intrinsic component of membrane	GO:0031224	1	**$4.49x10^{-2}$**

Supplementary Table 4. Over-representation analyses of Gene Ontology categories among *historical* common west-east differentially methylated genes (total of 699)

**Category Name**	**Accession N°**	**Adj. *P***
Ontology biological process		
single-multicellular organism process	GO:0044707	$6.62x10^{-15}$
multicellular organismal development	GO:0007275	$1.18x10^{-14}$
nervous system development	GO:0007399	$1.18x10^{-14}$
multicellular organismal process	GO:0032501	$1.99x10^{-14}$
developmental process	GO:0032502	$4.96x10^{-13}$
single-organism developmental process	GO:0044767	$8.45x10^{-13}$
system development	GO:0048731	$1.03x10^{-12}$
neuron differentiation	GO:0030182	$1.28x10^{-12}$
neurogenesis	GO:0022008	$2.03x10^{-11}$
generation of neurons	GO:0048699	$2.41x10^{-11}$
anatomical structure development	GO:0048856	$7.69x10^{-11}$
central nervous system development	GO:0007417	$7.33x10^{-10}$
organ development	GO:0048513	$3.01x10^{-9}$
cell differentiation	GO:0030154	$3.66x10^{-9}$
central nervous system neuron differentiation	GO:0021953	$1.26x10^{-8}$
cell development	GO:0048468	$1.76x10^{-8}$
cell fate commitment	GO:0045165	$2.25x10^{-8}$
cellular developmental process	GO:0048869	$4.17x10^{-8}$
tissue development	GO:0009888	$1.68x10^{-7}$
organ morphogenesis	GO:0009887	$2.32x10^{-7}$
anatomical structure morphogenesis	GO:0009653	$2.60x10^{-7}$
regulation of cell differentiation	GO:0045595	$2.60x10^{-7}$
brain development	GO:0007420	$2.60x10^{-7}$
regulation of neuron differentiation	GO:0045664	$2.75x10^{-7}$
cell-cell signaling	GO:0007267	$3.27x10^{-7}$
neuron fate commitment	GO:0048663	$7.03x10^{-7}$
regulation of multicellular organismal process	GO:0051239	$8.60x10^{-7}$
regulation of multicellular organismal development	GO:2000026	$1.68x10^{-6}$
positive regulation of developmental process	GO:0051094	$4.53x10^{-6}$
regulation of developmental process	GO:0050793	$5.11x10^{-6}$
embryo development	GO:0009790	$5.72x10^{-6}$
regulation of neurogenesis	GO:0050767	$7.43x10^{-6}$
positive regulation of cell differentiation	GO:0045597	$1.47x10^{-5}$
regulation of nervous system development	GO:0051960	$1.52x10^{-5}$
pattern specification process	GO:0007389	$2.25x10^{-5}$
muscle structure development	GO:0061061	$4.47x10^{-5}$
single-organism process	GO:0044699	$4.47x10^{-5}$
cell-cell adhesion	GO:0016337	$4.47x10^{-5}$
forebrain development	GO:0030900	$4.54x10^{-5}$
regulation of cell development	GO:0060284	$4.54x10^{-5}$
neuron development	GO:0048666	$5.49x10^{-5}$
synaptic transmission	GO:0007268	$7.67x10^{-5}$
spinal cord development	GO:0021510	$8.63x10^{-5}$

cell differentiation in spinal cord	GO:0021515	$9.01x10^{-5}$
neuron projection development	GO:0031175	$1.05x10^{-4}$
cell morphogenesis involved in differentiation	GO:0000904	$1.17x10^{-4}$
neuron projection morphogenesis	GO:0048812	$1.21x10^{-4}$
axonogenesis	GO:0007409	$1.82x10^{-4}$
epithelium development	GO:0060429	$1.86x10^{-4}$
ventral spinal cord development	GO:0021517	$3.09x10^{-4}$
tissue morphogenesis	GO:0048729	$3.79x10^{-4}$
forebrain neuron differentiation	GO:0021879	$5.02x10^{-4}$
cell morphogenesis involved in neuron differentiation	GO:0048667	$5.17x10^{-4}$
single-organism cellular process	GO:0044763	$5.17x10^{-4}$
cell adhesion	GO:0007155	$6.25x10^{-4}$
forebrain generation of neurons	GO:0021872	$6.73x10^{-4}$
biological adhesion	GO:0022610	$6.80x10^{-4}$
dorsal/ventral pattern formation	GO:0009953	$7.19x10^{-4}$
positive regulation of multicellular organismal process	GO:0051240	$8.90x10^{-4}$
positive regulation of neuron differentiation	GO:0045666	$8.90x10^{-4}$
regulation of calcium ion-dependent exocytosis	GO:0017158	$1.14x10^{-3}$
pallium development	GO:0021543	$1.18x10^{-3}$
axon development	GO:0061564	$1.22x10^{-3}$
cell surface receptor signaling pathway	GO:0007166	$1.27x10^{-3}$
regulation of secretion	GO:0051046	$1.29x10^{-3}$
neuron fate specification	GO:0048665	$1.33x10^{-3}$
tube development	GO:0035295	$1.36x10^{-3}$
embryonic morphogenesis	GO:0048598	$1.43x10^{-3}$
telencephalon development	GO:0021537	$1.43x10^{-3}$
cerebral cortex development	GO:0021987	$1.79x10^{-3}$
behavior	GO:0007610	$2.03x10^{-3}$
neuron migration	GO:0001764	$2.28x10^{-3}$
morphogenesis of an epithelium	GO:0002009	$2.37x10^{-3}$
neurotransmitter transport	GO:0006836	$2.65x10^{-3}$
regulation of neurotransmitter levels	GO:0001505	$2.65x10^{-3}$
locomotory behavior	GO:0007626	$2.73x10^{-3}$
homophilic cell adhesion	GO:0007156	$2.92x10^{-3}$
cell fate specification	GO:0001708	$2.99x10^{-3}$
regionalization	GO:0003002	$3.12x10^{-3}$
response to organic cyclic compound	GO:0014070	$3.26x10^{-3}$
locomotion	GO:0040011	$3.87x10^{-3}$
regulation of exocytosis	GO:0017157	$3.98x10^{-3}$
muscle tissue development	GO:0060537	$4.09x10^{-3}$
positive regulation of secretion	GO:0051047	$4.24x10^{-3}$
sensory organ development	GO:0007423	$4.31x10^{-3}$
striated muscle tissue development	GO:0014706	$4.48x10^{-3}$
negative regulation of neuron differentiation	GO:0045665	$4.48x10^{-3}$
axon guidance	GO:0007411	$4.48x10^{-3}$
neuron projection guidance	GO:0097485	$4.48x10^{-3}$
cell projection morphogenesis	GO:0048858	$4.57x10^{-3}$
response to endogenous stimulus	GO:0009719	$4.57x10^{-3}$
neuron-neuron synaptic transmission	GO:0007270	$4.59x10^{-3}$

response to steroid hormone	GO:0048545	$4.84x10^{-3}$
digestive system development	GO:0055123	$4.98x10^{-3}$
positive regulation of calcium ion-dependent exocytosis	GO:0045956	$5.65x10^{-3}$
anatomical structure formation involved in morphogenesis	GO:0048646	$5.90x10^{-3}$
calcium ion-dependent exocytosis	GO:0017156	$6.20x10^{-3}$
cell part morphogenesis	GO:0032990	$6.53x10^{-3}$
cell migration	GO:0016477	$6.56x10^{-3}$
cellular component movement	GO:0006928	$6.74x10^{-3}$
response to chemical	GO:0042221	$8.15x10^{-3}$
localization of cell	GO:0051674	$8.55x10^{-3}$
signaling	GO:0023052	$9.00x10^{-3}$
single organism signaling	GO:0044700	$9.00x10^{-3}$
monoamine transport	GO:0015844	$9.96x10^{-3}$
muscle cell differentiation	GO:0042692	$1.01x10^{-2}$
regulation of amine transport	GO:0051952	$1.05x10^{-2}$
single-organism behavior	GO:0044708	$1.06x10^{-2}$
response to external stimulus	GO:0009605	$1.06x10^{-2}$
neurotransmitter secretion	GO:0007269	$1.12x10^{-2}$
hindbrain development	GO:0030902	$1.12x10^{-2}$
signal release	GO:0023061	$1.18x10^{-2}$
gland development	GO:0048732	$1.18x10^{-2}$
response to lipid	GO:0033993	$1.20x10^{-2}$
neurological system process	GO:0050877	$1.23x10^{-2}$
negative regulation of developmental process	GO:0051093	$1.23x10^{-2}$
lung development	GO:0030324	$1.25x10^{-2}$
cell communication	GO:0007154	$1.27x10^{-2}$
cellular component morphogenesis	GO:0032989	$1.29x10^{-2}$
cell morphogenesis	GO:0000902	$1.32x10^{-2}$
embryonic organ development	GO:0048568	$1.42x10^{-2}$
chemotaxis	GO:0006935	$1.42x10^{-2}$
taxis	GO:0042330	$1.42x10^{-2}$
G-protein coupled receptor signaling pathway	GO:0007186	$1.45x10^{-2}$
respiratory tube development	GO:0030323	$1.46x10^{-2}$
dopamine transport	GO:0015872	$1.47x10^{-2}$
cell projection organization	GO:0030030	$1.48x10^{-2}$
digestive tract development	GO:0048565	$1.75x10^{-2}$
spinal cord motor neuron differentiation	GO:0021522	$1.83x10^{-2}$
response to cocaine	GO:0042220	$1.88x10^{-2}$
nitric oxide mediated signal transduction	GO:0007263	$1.94x10^{-2}$
amine transport	GO:0015837	$1.98x10^{-2}$
cell motility	GO:0048870	$2.06x10^{-2}$
multicellular organismal response to stress	GO:0033555	$2.16x10^{-2}$
response to alcohol	GO:0097305	$2.43x10^{-2}$
pituitary gland development	GO:0021983	$2.52x10^{-2}$
positive regulation of exocytosis	GO:0045921	$2.52x10^{-2}$
neuropeptide signaling pathway	GO:0007218	$2.76x10^{-2}$
secretion	GO:0046903	$2.80x10^{-2}$
regulation of stem cell proliferation	GO:0072091	$2.83x10^{-2}$
organ formation	GO:0048645	$2.85x10^{-2}$

stem cell differentiation	GO:0048863	$3.27x10^{-2}$
striated muscle cell differentiation	GO:0051146	$3.28x10^{-2}$
negative regulation of cell differentiation	GO:0045596	$3.46x10^{-2}$
secretion by cell	GO:0032940	$3.46x10^{-2}$
respiratory system development	GO:0060541	$3.46x10^{-2}$
exocrine system development	GO:0035272	$3.46x10^{-2}$
muscle organ development	GO:0007517	$3.46x10^{-2}$
developmental induction	GO:0031128	$3.68x10^{-2}$
cellular response to endogenous stimulus	GO:0071495	$3.74x10^{-2}$
neurotransmitter uptake	GO:0001504	$3.77x10^{-2}$
developmental growth	GO:0048589	$3.80x10^{-2}$
synaptic transmission, glutamatergic	GO:0035249	$4.13x10^{-2}$
cell-cell signaling involved in cell fate commitment	GO:0045168	$4.30x10^{-2}$
epithelial tube branching involved in lung morphogenesis	GO:0060441	$4.37x10^{-2}$
regulation of postsynaptic membrane potential	GO:0060078	$4.38x10^{-2}$
regulation of transport	GO:0051049	$4.78x10^{-2}$
regulation of localization	GO:0032879	$4.92x10^{-2}$
Ontology molecular function		
sequence-specific DNA binding	GO:0043565	$3.25x10^{-9}$
nucleic acid binding transcription factor activity	GO:0001071	$8.80x10^{-7}$
sequence-specific DNA binding transcription factor activity	GO:0003700	$1.65x10^{-6}$
transmembrane signaling receptor activity	GO:0004888	$1.52x10^{-5}$
signaling receptor activity	GO:0038023	$4.47x10^{-5}$
receptor activity	GO:0004872	$1.48x10^{-4}$
calcium ion binding	GO:0005509	$7.02x10^{-4}$
signal transducer activity	GO:0004871	$8.89x10^{-4}$
molecular transducer activity	GO:0060089	$8.89x10^{-4}$
transcription regulatory region sequence-specific DNA binding	GO:0000976	$2.09x10^{-3}$
transcription regulatory region DNA binding	GO:0044212	$4.07x10^{-3}$
regulatory region DNA binding	GO:0000975	$5.90x10^{-3}$
regulatory region nucleic acid binding	GO:0001067	$5.90x10^{-3}$
G-protein coupled receptor activity	GO:0004930	$6.27x10^{-3}$
transmembrane receptor protein kinase activity	GO:0019199	$3.80x10^{-2}$
growth factor binding	GO:0019838	$3.91x10^{-2}$
Ontology cellular component		
integral component of plasma membrane	GO:0005887	2.41x10-11
intrinsic component of plasma membrane	GO:0031226	6.42x10-11
plasma membrane part	GO:0044459	$4.17x10^{-8}$
integral component of membrane	GO:0016021	$6.67x10^{-6}$
intrinsic component of membrane	GO:0031224	$1.20x10^{-5}$
cell periphery	GO:0071944	$4.47x10^{-5}$
plasma membrane	GO:0005886	$5.15x10^{-5}$
membrane part	GO:0044425	$8.14x10^{-4}$
ion channel complex	GO:0034702	$2.78x10^{-2}$
synaptic vesicle	GO:0008021	$3.74x10^{-2}$

Supplementary Table 5. Over-representation analyses of Gene Ontology categories among *recent* and *historical* differentially methylated genes (total of 2,145 and 1,507, respectively) using the filtered dataset, which excludes 51,386 immune cell-associated methylation sites. Values in bold correspond to FDR-adjusted significant *P*-values.

**Category Name**	**Accession N°**	**FDR-Adjusted *P***	
		Recent	***Historical***
Ontology biological process			
multi-organism cellular process	GO:0044764	**3.86×10^{-5}**	1
viral process	GO:0016032	**6.57×10^{-5}**	1
interspecies interaction between organisms	GO:0044419	**8.11×10^{-5}**	1
symbiosis, encompassing mutualism through parasitism	GO:0044403	**8.11×10^{-5}**	1
intracellular protein transport	GO:0006886	**1.64×10^{-4}**	1
protein transport	GO:0015031	**2.04×10^{-4}**	1
establishment of protein localization	GO:0045184	**2.88×10^{-4}**	1
protein localization	GO:0008104	**3.17×10^{-3}**	1
macromolecule localization	GO:0033036	**4.17×10^{-3}**	1
cellular macromolecule localization	GO:0070727	**5.32×10^{-3}**	1
cellular protein localization	GO:0034613	**5.76×10^{-3}**	1
translational termination	GO:0006415	**5.76×10^{-3}**	NA
intracellular transport	GO:0046907	**1.04×10^{-2}**	1
protein targeting to ER	GO:0045047	**1.23×10^{-2}**	NA
protein localization to endoplasmic reticulum	GO:0070972	**1.55×10^{-2}**	NA
RNA catabolic process	GO:0006401	**1.56×10^{-2}**	1
establishment of protein localization to endoplasmic reticulum	GO:0072599	**1.56×10^{-2}**	NA
single-organism intracellular transport	GO:1902582	**1.64×10^{-2}**	1
SRP-dependent cotranslational protein targeting to membrane	GO:0006614	**1.72×10^{-2}**	NA
single-organism cellular localization	GO:1902580	**2.01×10^{-2}**	1
single-organism localization	GO:1902578	**2.01×10^{-2}**	1
organelle organization	GO:0006996	**2.03×10^{-2}**	1
mRNA catabolic process	GO:0006402	**2.13×10^{-2}**	1
mRNA metabolic process	GO:0016071	**2.13×10^{-2}**	1
translational elongation	GO:0006414	**2.13×10^{-2}**	1
cotranslational protein targeting to membrane	GO:0006613	**2.13×10^{-2}**	NA
nuclear-transcribed mRNA catabolic process	GO:0000956	**2.69×10^{-2}**	1
protein targeting	GO:0006605	**3.23×10^{-2}**	1
cytoplasmic transport	GO:0016482	**4.49×10^{-2}**	1
multicellular organismal development	GO:0007275	1	**7.01×10^{-5}**
single-multicellular organism process	GO:0044707	1	**7.01×10^{-5}**
cell fate commitment	GO:0045165	1	**1.07×10^{-4}**
multicellular organismal process	GO:0032501	1	**1.07×10^{-4}**
single-organism developmental process	GO:0044767	1	**1.87×10^{-4}**
developmental process	GO:0032502	1	**2.72×10^{-4}**
system development	GO:0048731	1	**5.86×10^{-4}**
synaptic transmission	GO:0007268	1	**6.87×10^{-4}**

neuron differentiation	GO:0030182	1	**$1.40x10^{-3}$**
nervous system development	GO:0007399	1	**$1.91x10^{-3}$**
neurogenesis	GO:0022008	1	**$1.91x10^{-3}$**
central nervous system development	GO:0007417	1	**$1.96x10^{-3}$**
cell-cell signaling	GO:0007267	1	**$2.00x10^{-3}$**
anatomical structure development	GO:0048856	1	**$3.80x10^{-3}$**
organ development	GO:0048513	1	**$4.83x10^{-3}$**
neuron fate commitment	GO:0048663	1	**$5.94x10^{-3}$**
generation of neurons	GO:0048699	1	**$6.42x10^{-3}$**
cell differentiation	GO:0030154	1	**$6.64x10^{-3}$**
cellular developmental process	GO:0048869	1	**$1.13x10^{-2}$**
organ morphogenesis	GO:0009887	1	**$1.49x10^{-2}$**
regulation of cell differentiation	GO:0045595	1	**$1.97x10^{-2}$**
Ontology molecular function			
poly(A) RNA binding	GO:0044822	**$6.82x10^{-6}$**	1
RNA binding	GO:0003723	**$2.04x10^{-4}$**	1
protein binding	GO:0005515	**$1.04x10^{-2}$**	1
organic cyclic compound binding	GO:0097159	**$3.35x10^{-2}$**	1
heterocyclic compound binding	GO:1901363	**$3.81x10^{-2}$**	1
metal ion transmembrane transporter activity	GO:0046873	1	**$2.25x10^{-2}$**
sequence-specific DNA binding	GO:0043565	1	**$2.27x10^{-2}$**
Ontology cellular component			
cytoplasm	GO:0005737	**$2.29x10^{-10}$**	1
intracellular	GO:0005622	**$2.51x10^{-10}$**	1
intracellular part	GO:0044424	**$3.21x10^{-10}$**	1
intracellular organelle part	GO:0044446	**$1.20x10^{-9}$**	1
intracellular organelle	GO:0043229	**$1.49x10^{-9}$**	1
organelle	GO:0043226	**$5.92x10^{-9}$**	1
organelle part	GO:0044422	**$8.97x10^{-9}$**	1
intracellular membrane-bounded organelle	GO:0043231	**$2.10x10^{-8}$**	1
membrane-bounded organelle	GO:0043227	**$1.04x10^{-6}$**	1
cytoplasmic part	GO:0044444	**$1.65x10^{-6}$**	1
nucleus	GO:0005634	**$8.45x10^{-4}$**	1
intracellular non-membrane-bounded organelle	GO:0043232	**$1.04x10^{-2}$**	1
non-membrane-bounded organelle	GO:0043228	**$1.04x10^{-2}$**	1
organelle membrane	GO:0031090	**$1.04x10^{-2}$**	1
ribonucleoprotein complex	GO:0030529	**$1.23x10^{-2}$**	1
ruffle	GO:0001726	**$1.23x10^{-2}$**	1
cytosol	GO:0005829	**$1.72x10^{-2}$**	1
nuclear part	GO:0044428	**$2.69x10^{-2}$**	1
integral component of plasma membrane	GO:0005887	1	**$4.41x10^{-4}$**
intrinsic component of plasma membrane	GO:0031226	1	**$6.87x10^{-4}$**
plasma membrane part	GO:0044459	1	**$1.40x10^{-3}$**
integral component of membrane	GO:0016021	1	**$8.02x10^{-3}$**
intrinsic component of membrane	GO:0031224	1	**$1.62x10^{-2}$**
ion channel complex	GO:0034702	1	**$2.47x10^{-2}$**
membrane part	GO:0044425	1	**$4.29x10^{-2}$**

Supplementary Table 6. Population specificity of African meQTLs. Genome-wide estimation of the weight of each population combination, estimated by the eQtlBma model used for meQTL mapping.

Configuration	Weight
e-AGR	$6.52x10^{-16}$
e-RHG	0.233
f-AGR	$6.19x10^{-18}$
w-AGR	$2.01x10^{-7}$
w-RHG	0.121
e-AGR + e-RHG	$3.97x10^{-7}$
e-AGR + f-AGR	$9.38x10^{-20}$
e-AGR + w-AGR	$1.03x10^{-10}$
e-AGR + w-RHG	$4.62x10^{-10}$
e-RHG + f-AGR	$2.77x10^{-13}$
e-RHG + w-AGR	$4.96x10^{-4}$
e-RHG + w-RHG	$2.11x10^{-2}$
f-AGR + w-AGR	$3.18x10^{-11}$
f-AGR + w-RHG	$6.87x10^{-4}$
w-AGR + w-RHG	$6.71x10^{-3}$
e-AGR + e-RHG + f-AGR	$4.90x10^{-16}$
e-AGR + e-RHG + w-AGR	$6.39x10^{-4}$
e-AGR + e-RHG + w-RHG	$1.40x10^{-8}$
e-AGR + f-AGR + w-AGR	$3.35x10^{-3}$
e-AGR + f-AGR + w-RHG	$6.76x10^{-9}$
e-AGR + w-AGR + w-RHG	$1.62x10^{-6}$
e-RHG + f-AGR + w-AGR	$7.45x10^{-12}$
e-RHG + f-AGR + w-RHG	$8.86x10^{-5}$
e-RHG + w-AGR + w-RHG	$1.23x10^{-4}$
f-AGR + w-AGR + w-RHG	$8.52x10^{-3}$
e-AGR + e-RHG + f-AGR + w-AGR	$1.48x10^{-2}$
e-AGR + e-RHG + f-AGR + w-RHG	$1.25x10^{-16}$
e-AGR + e-RHG + w-AGR + w-RHG	$1.67x10^{-10}$
e-AGR + f-AGR + w-AGR + w-RHG	$4.50x10^{-2}$
e-RHG + f-AGR + w-AGR + w-RHG	$6.93x10^{-5}$
e-AGR + e-RHG + f-AGR + w-AGR + w-RHG	$5.44x10^{-1}$

Supplementary Table 7. Primer sequences used for bisulfite PCR-pyrosequencing

IGF2BP2_cg23956648 F: /5Biosg/TAT TTA GGG GTT TTG GGT TGA TGT T<br>IGF2BP2_cg23956648 R: CCC CCA ATA AAA TAT CTT AAA TAA CAC T<br>IGF2BP2_cg23956648 S: ACT TAA AAT AAA AAT AAT TCT TCA CAC
HOXC6_cg21582112 F: ATG TAA TAG AAA AAG GAG AAA AAG AAG TA<br>HOXC6_cg21582112 R: /5Biosg/ACC CTC ACT AAC TCC AAA TAA TAA<br>HOXC6_cg21582112 S: GTA GGG AGG TGG TAT
ZNF492_cg09314196 F: TAG AGT GGG AAA TAA AGT GAG ATT ATG T<br>ZNF492_cg09314196 R: /5Biosg/CTC AAA TAA TCC ATC CTC CTT AAC<br>ZNF492_cg09314196 S: TGT AGT AGA ATG AAA TGT TTT T
Enhancer_cg23053977 F: GGG GTA TAG TAG AAG AAA ATT TTA AGG<br>Enhancer_cg23053977 R: /5BiodT/TT TTC CCA CAA TAC AAC TAA ATA TTC AC<br>Enhancer_cg23053977 S: TTT TTA GAA TAA AGT AAG TAT TTA ATG AT
DOCK1_cg06406458 F: AGT TAT TTT TTT TTG TTA TTT TGG GTG AA<br>DOCK1_cg06406458 R: /5Biosg/TCA ATA CTT TTC CCT ATT TCT CTT ATC AC<br>DOCK1_cg06406458 S: TGA AAA AAG GGA TGA TAT GTT TAG
COL23A1_ cg08684511 F: GGT GTT TGT AGT TTA AGG GTA TGT AG<br>COL23A1_ cg08684511 R: /5BiodT/CA ACT AAA AAC TAA CAC CAT ATA CCT<br>COL23A1_ cg08684511 S: TGA GGA GTG AAA TTG TAT TTA ATT AT
RORA_ cg09879458 F: GAT TAT ATT TTG TGG GGT GAA TGG AGG TA<br>RORA_ cg09879458 R: /5BiodT/CC TCC CAA CCT TTA TTA TTC CTT TTC C<br>RORA_ cg09879458 S: AGT AAT ATA TAG TAG TAT GAG AAA T
ADAM28_ cg18757155 F: GAG TAT GGT AAA GGA GAG TAA AAT AAT AGT<br>ADAM28_ cg18757155 R: /5BiodT/AA ATA AAA CTC CCT AAT TCA TTC TTA TCT<br>ADAM28_ cg18757155 S: GGA GGT AGT TAG GAT TT

Supplementary Notes

Supplementary Note 1. Different Methylation Profiles across Genomic Regions
Individual methylation data showed the expected bimodal profiles (Supplementary Fig. 1a), with M-values ranging from -6 to +6 and two peaks at -3.5 and +3.5, corresponding to unmethylated sites and fully methylated sites, respectively. The level of methylation decreased near and within CpG islands (Supplementary Fig. 1b). We observed a lower level of methylation at sites around the transcription start sites of genes than in intergenic and gene body regions (Supplementary Fig. 1c). In addition, sites in gene bodies were slightly more methylated than intergenic sites, as previously reported[2]. Finally, when looking at inter-individual variation in methylation levels, we observed that the correlation between methylation profiles of technical replicates (19 replicates in total) was higher than the correlation between randomly chosen pairs of samples from the same population (Supplementary Fig. 1d). Samples from w-AGR and e-AGR populations, which showed stronger correlations between pairs of samples, tended to be more homogeneous than samples from f-AGR, w-RHG and e-RHG.

Supplementary Note 2. Age Prediction from Methylation Data
As age has been shown to impact on the variation of DNA methylation[3], we controlled for this potential bias in our DMS and meQTL analyses. Ages were available for all except one samples from the western setting, i.e., 256 individuals from w-RHG, w-AGR and f-AGR populations of the Gabon/Cameroon region, and for none of those from the eastern setting, i.e., 95 individuals from e-RHG and e-AGR populations of Uganda. We thus estimated ages for all samples, using an elastic net regression method[1], and compared predicted with declared ages, when the latter were available. Although predicted ages were, on average, ~7 years older than declared ages, we observed a high correlation between the two, with a Pearson’s correlation coefficient of 0.84 (Supplementary Fig. 2). Moreover, samples from all three populations showed the same skew in predicted ages, so the relative ranks of ages were conserved between declared and predicted ages. Given the relative accuracy of the predictive model, and for the sake of consistency between comparisons, we used the predicted ages for all samples as a covariate in the linear regressions performed for the DMS and meQTL analyses.

Supplementary Note 3. Accounting for Heterogeneity in Blood Cell Composition
Because environmental factors could alter the relative abundance of different cell types in the blood of the populations studied, and thus confound our analysis[4], we predicted the proportions of different cells types in unfractionated whole blood, as previously described[5] (Methods). We found that our predictions fall within generally accepted ranges: 47% for granulocytes, 11% for $CD8^+$-T cells, 14% for $CD4^+$-T cells, 12% for NK cells, 10% for B

cells and 6% for monocytes. To evaluate the accuracy of the predictive model, we compared these estimations with fluorescence-activated cell sorting (FACS) data from peripheral blood mononuclear cells (PBMCs), which were obtained in 35 e-RHG and 31 e-AGR. Our results showed high correlation coefficients (Pearson's R: 0.48-0.57) between estimated and observed proportions for the cellular types from which we could obtain FACS data ($CD4^+$ T-cells, $CD8^+$ T-cells, B-cells, and NK-cells) (Supplementary Fig. 3). In light of this, we used the estimated cell subtype proportions, together with gender and age, to adjust M-values for all subsequent analyses, including principal component analyses (PCA), the estimation of DMS and the mapping of meQTLs.

We assessed the efficiency of this correction by examining the extent to which the different PCs of the PCA were correlated with variation in blood cell proportions. While age and cell counts strongly correlated with the first ten PCs using unadjusted M-values (Supplementary Table 1, Supplementary Fig. 4a), the subsistence strategy (RHG *vs*. AGR) and geographic location (western *vs*. eastern central Africa) of the populations were the only factors associated with the first ten PCs using adjusted M-values (Supplementary Table 1, Supplementary Fig. 4b). In addition, we performed a PCA of unadjusted M-values in 35 e-RHG and 31 e-AGR, for which FACS data could be obtained, and indeed observed significant associations between *measured* cell type proportions and the first eight PCs (accounting for 40% of the total variance explained). Importantly, when adjusting M-values for age, sex and *predicted* cell counts, we observed no association between the first eight PCs (accounting for 31% of the total variance explained) and *experimentally-measured* cell type proportions ($P > 0.05$). This clearly indicates that correcting our data using predicted cell types should adjust DNA methylation values for potential variation in cell type proportions between populations.

Supplementary Note 4. Replication based on a filtered dataset for cell type-associated methylation probes

Given the importance of accounting for blood cell heterogeneity[4], and because our adjustments might have not fully corrected for this possible confounder, we evaluated the extent to which our main findings may be affected by variation in blood cell proportions. First, we verified if there were differences in predicted immune cell counts across the studied populations. No significant differences were observed in the f-AGR/w-AGR comparison (used to identify *recent* DMS), with the exception of an increased number of monocytes in w-AGR (P=2.9x10^{-7}, Supplementary Fig. 8a). Likewise, no significant differences were detected in the f-AGR/w-RHG population comparison (i.e., used to identify *historical* DMS), with the exception of a slight increased number of $CD4^+$ T-cells in f-AGR (P=0.013, Supplementary Fig. 8b). Nevertheless, both $CD4^+$ T-cells and monocytes represent 14% and 6% of all whole blood cells, respectively, a small fraction

that is not expected to have a major impact on the global patterns of DNA methylation variation across populations.

To evaluate further the impact of the use of whole blood, we sought to confirm the main findings of our study using a filtered dataset, in which we applied a highly conservative correction, i.e., we removed all probes whose methylation state is known to vary across immune cell subtypes. We thus excluded a total of 51,386 HumanMethylation450 probes, which have been shown to be associated with any immune cell type ($P < 0.01$, after a Bonferroni correction) in the study of Jaffe and colleagues[4].

Using this filtered dataset, we identified 16,814 DMS between w-RHG and w-AGR (34.8% reduction compared to the global analyses using the adjusted, unfiltered dataset), 14,221 DMS between e-RHG and e-AGR (26.7% reduction), 2,968 *recent* DMS (48.1% reduction) and 2,818 *historical* DMS (30.4% reduction), using a FDR < 0.01. We next tested if differentially methylated genes in *recent* and *historical* DMS sets were enriched in gene ontology (GO) categories. We found that genes containing *recent* DMS were enriched in categories related to immunity to infection, host-pathogen interactions and various cellular processes, including “viral process”, “symbiosis, encompassing mutualism through parasitism” and “interspecies interaction between organisms” (adjusted $P < 1x10^{-4}$; Supplementary Table 5). Genes containing *historical* DMS were enriched in categories mostly related to development, such as “multicellular organismal development” and “developmental process” (adjusted $P < 5x10^{-4}$; Supplementary Table 5). The results of GO enrichment analyses, using the filtered dataset, are thus in good agreement with those obtained using the global dataset, and show that the biological functions associated with *recent* and *historical* DMS clearly differ.

Finally, using the filtered dataset, *historical* DMS were strongly enriched in meQTLs (27%, OR=3.7, SE=0.04; resampling $P < 10^{-7}$), whereas *recent* DMS were depleted in such genetic associations (7%, OR=0.7, SE=0.07; resampling $P < 10^{-7}$). These results confirm that *historical* DMS are more strongly associated with genotypic differences than the remaining sets of DMS. Taken together, our adjustments for cell proportions (Supplementary Note 3), together with the measures we adopted to control for this possible confounding, strongly support the notion that variability in blood cell subtypes does not have a major effect in our main conclusions.

Supplementary Note 5. Population specificity of meQTLs in whole blood.
Fraser and colleagues[6] compared CEU and YRI populations from the HapMap Project, using the HumanMethylation27 BeadChip assay on lymphoblastoid cell lines (LCLs). They found that only 8.9% of detected meQTLs were shared between the two populations,

consistent with the extensive population specificity of DNA methylation heritability estimates. Moen and colleagues[2] compared the same populations and cell lines, but used the HumanMethylation450 BeadChip assay. To illustrate the extent of population specificity of their meQTLs, they reported a very low correlation (−0.03) between SNP association R^2 estimated in CEU and YRI, suggesting that genetic epistasis and/or G×E interactions could be common in explaining DNA methylation variation[2]. In our study, we estimated that 90% of meQTLs are shared across populations. To test whether this proportion could be accounted for by a bias of eQtlBma against population-specific meQTL associations, we reanalyzed our data using the approach of Moen and colleagues[2]. We estimated R^2 coefficients of a linear regression model of DNA methylation on SNP genotypes, correcting for age, gender, ancestry and immune cell proportions. We obtained strong correlations (0.39 to 0.66) between association R^2 estimated in RHG and AGR populations (Supplementary Fig. 10). This result attests that eQtlBma is not biased against population-specific meQTL associations, and that most meQTLs detected here are indeed shared among populations. We suggest that this pattern result from (i) the lower degree of genetic differentiation of our populations (F_{ST}=0.023-0.032) with respect to CEU and YRI (F_{ST}=0.12)[7], and (ii) DNA methylation was assessed here in whole blood, a complex mixture of different cell types, while the studies mentioned above used LCLs.

Supplementary References:

1 Horvath, S. DNA methylation age of human tissues and cell types. *Genome Biol* **14**, R115 (2013).

2 Moen, E. L. *et al.* Genome-wide variation of cytosine modifications between European and African populations and the implications for complex traits. *Genetics* **194**, 987-996 (2013).

3 Bell, J. T. *et al.* Epigenome-wide scans identify differentially methylated regions for age and age-related phenotypes in a healthy ageing population. *PLoS Genet* **8**, e1002629 (2012).

4 Jaffe, A. E. & Irizarry, R. A. Accounting for cellular heterogeneity is critical in epigenome-wide association studies. *Genome Biol* **15**, R31 (2014).

5 Houseman, E. A. *et al.* DNA methylation arrays as surrogate measures of cell mixture distribution. *BMC Bioinformatics* **13**, 86 (2012).

6 Fraser, H. B., Lam, L. L., Neumann, S. M. & Kobor, M. S. Population-specificity of human DNA methylation. *Genome Biol* **13**, R8 (2012).

7 Abecasis, G. R. *et al.* An integrated map of genetic variation from 1,092 human genomes. *Nature* **491**, 56-65 (2012).
